# Metabolic engineering of *Streptomyces peucetius* for biosynthesis of *N,N*-dimethylated anthracyclines

**DOI:** 10.3389/fbioe.2024.1363803

**Published:** 2024-02-28

**Authors:** Mandy B. Hulst, Le Zhang, Helga U. van der Heul, Chao Du, Somayah S. Elsayed, Arina Koroleva, Thadee Grocholski, Dennis P. A. Wander, Mikko Metsä-Ketelä, Jacques J. C. Neefjes, Gilles P. van Wezel

**Affiliations:** ^1^ Institute of Biology, Leiden University, Leiden, Netherlands; ^2^ Department of Life Technologies, University of Turku, Turku, Finland; ^3^ Department of Cell and Chemical Biology, ONCODE Institute, Leiden University Medical Centre LUMC, Leiden, Netherlands

**Keywords:** doxorubicin, anthracyclines, anticancer, metabolic engineering, biosynthesis, *Streptomyces*

## Abstract

**Introduction:** Daunorubicin and doxorubicin, two anthracycline polyketides produced by *S. peucetius*, are potent anticancer agents that are widely used in chemotherapy, despite severe side effects. Recent advances have highlighted the potential of producing improved derivatives with reduced side effects by incorporating l-rhodosamine, the *N,N*-dimethyl analogue of the native amino sugar moiety.

**Method:** In this study, we aimed to produce *N,N*-dimethylated anthracyclines by engineering the doxorubicin biosynthetic pathway in the industrial *Streptomyces peucetius* strain G001. To achieve this, we introduced genes from the aclarubicin biosynthetic pathway encoding the sugar *N*-methyltransferases AclP and AknX2. Furthermore, the native gene for glycosyltransferase DnrS was replaced with genes encoding the aclarubicin glycosyltransferases AknS and AknT. Additionally, the gene for methylesterase RdmC from the rhodomycin biosynthetic pathway was introduced.

**Results:** A new host was engineered successfully, whereby genes from the aclarubicin pathway were introduced and expressed. LC-MS/MS analysis of the engineered strains showed that dimethylated sugars were efficiently produced, and that these were incorporated ino the anthracycline biosynthetic pathway to produce the novel dimethylated anthracycline *N,N*-dimethyldaunorubicin. Further downstream tailoring steps catalysed by the cytochrome P450 monooxygenase DoxA exhibited limited efficacy with *N,N*-dimethylated substrates. This resulted in only low production levels of *N,N*-dimethyldaunorubicin and no *N,N*-dimethyldoxorubicin, most likely due to the low affinity of DoxA for dimethylated substrates.

**Discussion:** *S. peucetius* G001 was engineered such as to produce *N,N*-dimethylated sugars, which were incorporated into the biosynthetic pathway. This allowed the successful production of *N,N*-dimethyldaunorubicin, an anticancer drug with reduced cytotoxicity. DoxA is the key enzyme that determines the efficiency of the biosynthesis of *N,N*-dimethylated anthracyclines, and engineering of this enzyme will be a major step forwards towards the efficient production of more *N,N*-dimethylated anthracyclines, including *N,N*-dimethyldoxorubicin. This study provides valuable insights into the biosynthesis of clinically relevant daunorubicin derivatives, highlighting the importance of combinatorial biosynthesis.

## 1 Introduction

The secondary metabolic pathways of bacteria and fungi yield valuable natural products that serve as an important source of antibiotics and other drugs. Actinobacteria, especially members of the *Streptomyces* genus, stand out as prolific producers of these bioactive secondary metabolites (Bérdy, 2005; Barka et al., 2016). These compounds exhibit a broad range of bioactivities, including antibacterial, anticancer, antifungal, antiviral, anthelmintic, herbicidal, and immunosuppressive effects (Hopwood, 2007; Newman and Cragg, 2020). Natural products can be categorised into distinct structural families based on their biosynthetic origin, such as polyketides, non-ribosomal peptides, ribosomally synthesised post-translationally modified peptides (RiPPs), terpenoids, and alkaloids. The wide range of bioactivities and structural variations highlights the importance of natural products for drug development (Newman and Cragg, 2020).

**FIGURE 1:**
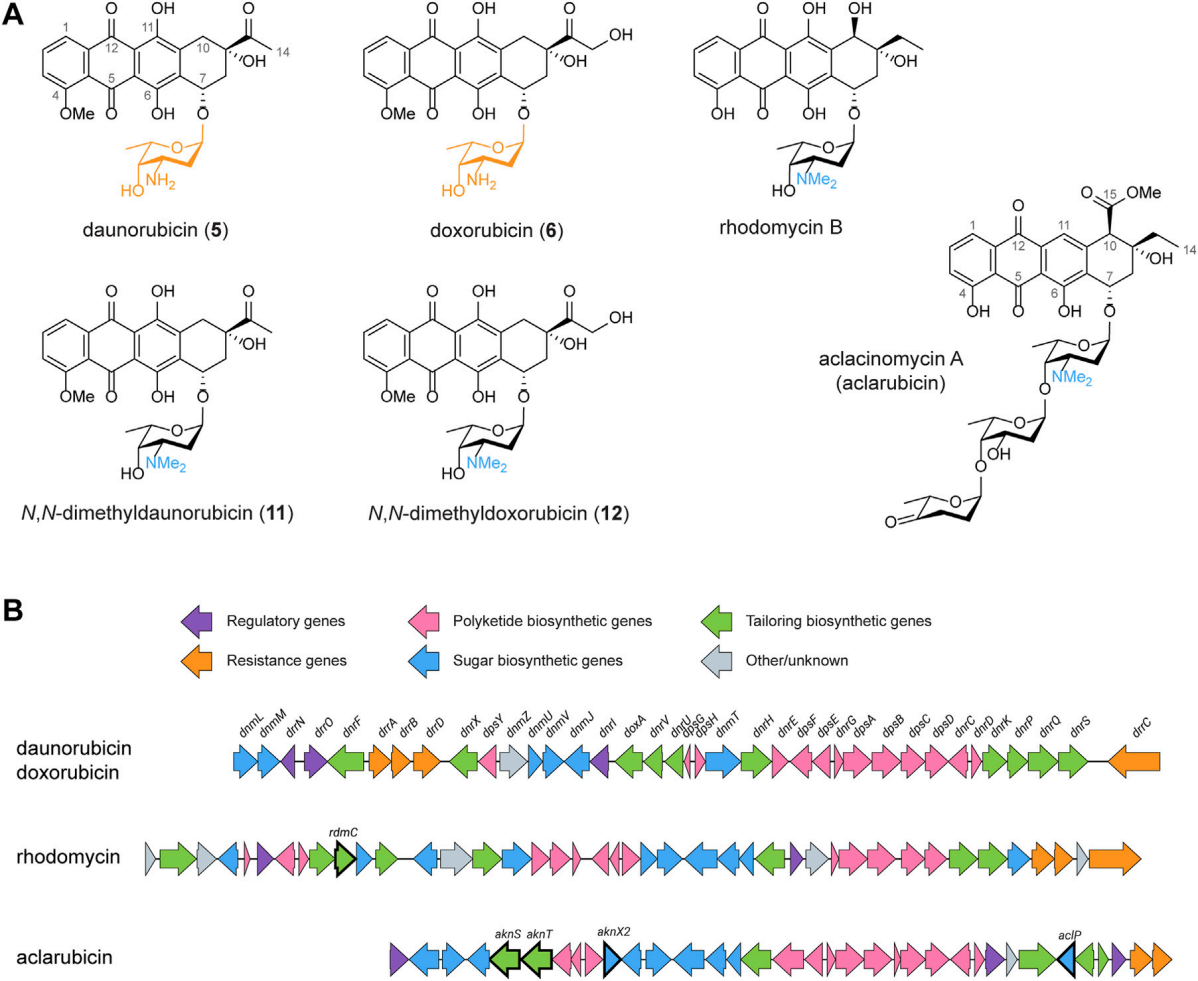
Chemical structures and BGCs of anthracyclines described in this work. **(A)** Chemical structures of daunorubicin, doxorubicin, *N*,*N*-dimethyldaunorubicin (**11**), *N*,*N*-dimethyldoxorubicin (**12**), rhodomycin B and aclacinomycin A (aclarubicin). **(B)** The BGCs of daunorubicin/doxorubicin, rhodomycin and aclarubicin were aligned and visualised using clinker (Gilchrist and Chooi, 2021). To achieve biosynthesis of *N*,*N*-dimethyldaunorubicin (**11**) and *N*,*N*-dimethyldoxorubicin (**12**), several genes from the rhodomycin and aclarubicin BGCs were introduced to *Streptomyces peucetius*, as highlighted in bold.

Polyketides are a diverse class of natural products renowned for their remarkable structural complexity (Hertweck, 2009). The polyketide backbone is assembled through the iterative condensation of acyl-CoA units, a process catalysed by the polyketide synthase (PKS) enzyme complexes (Staunton and Weissman, 2001). Type I polyketides are synthesised by large multimodular enzyme sets, while type II polyketides are synthesised by the iterative action of a single enzyme set (Shen, 2003). The polyketide scaffold is diversified by modifications introduced via tailoring reactions such as methylation, amination, oxidation, and glycosylation, resulting in a broad range of structures and biological activities (Olano et al., 2010). Anthracyclines are glycoside antibiotics whose aglycones are called anthracyclinones (Brockmann, 1963). They are aromatic type II polyketides that feature a linear tetracyclic 7,8,9,10-tetrahydro-5,12-naphthacenequinone scaffold and are decorated with one or more sugar moieties anthracyclinones (Brockmann, 1963). Anthracyclines are especially renowned for their potent anticancer activities (Hulst et al., 2022). The best-known members of this group, daunorubicin **5**) and doxorubicin **6**), are natural products of *Streptomyces peucetius* var. *caesius* (Camerino and Palamidessi, 1960; Arcamone et al., 1969; Di Marco et al., 1981). Daunorubicin **5**) and doxorubicin **6**) are glycosides of the amino sugar l-daunosamine (highlighted in orange in Figure 1A). These compounds have demonstrated exceptional efficacy against acute leukaemia and various types of solid tumours (Tan et al., 1967; Lown, 1993). Despite their clinical successes, the application of the drugs is limited by serious side effects, such as cardiotoxicity, therapy-related tumours and infertility (Weiss, 1992).

Anthracyclines have long been recognised as topoisomerase II inhibitors that induce DNA double-strand breaks (Frederick et al., 1990). However, a secondary effect of anthracyclines was recently discovered: the eviction of histones, which results in chromatin damage (Pang et al., 2013; van der Zanden et al., 2021). Notably, anthracyclines that trigger both, DNA double-strand breaks and histone eviction, are associated with cardiotoxicity, one of the major side effects of anthracycline drugs (Qiao et al., 2020; Wander et al., 2020; Wander et al., 2021; van Gelder et al., 2023). A screening of chemically synthesised doxorubicin derivatives resulted in a set of compounds with improved activities compared to doxorubicin (Qiao et al., 2020; Wander et al., 2020; Wander et al., 2021; van Gelder et al., 2023). Particularly, *N,N*-dimethylation of the amino sugar moiety results in the loss of DNA damage activity. *N,N*-dimethyldaunorubicin (**11**, Figure 1A) and *N,N*-dimethyldoxorubicin (**12**, Figure 1A) exhibit histone eviction activity without causing DNA damage, making them promising alternatives for anticancer treatment with reduced risk of cardiotoxicity (Qiao et al., 2020; van Gelder et al., 2023).

To achieve a sustainable and efficient production process for *N,N*-dimethyldaunorubicin (**11**) and *N,N*-dimethyldoxorubicin (**12**), biosynthesis presents a compelling alternative to chemical synthesis. Therefore, the aim of this work is to establish a biosynthetic production pathway for *N,N*-dimethyldaunorubicin (**11**) and *N,N*-dimethyldoxorubicin (**12**) in *S. peucetius*. Although *N,N*-dimethylated daunorubicin or doxorubicin have never been isolated from natural sources, the *N,N*-dimethylated amino sugar l-rhodosamine commonly occurs in natural anthracyclines, such as aclacinomycins (Figure 1A), rhodomycins (Figure 1A), cosmomycins, and cytorhodins (Metsä-Ketelä et al., 2008). Therefore, the doxorubicin biosynthetic pathway could potentially be modified for production of *N,N*-dimethyldaunorubicin (**11**) and *N,N*-dimethyldoxorubicin (**12**) by heterologous expression of genes from other anthracycline biosynthetic gene clusters (BGCs, Figure 1B).

**FIGURE 2:**
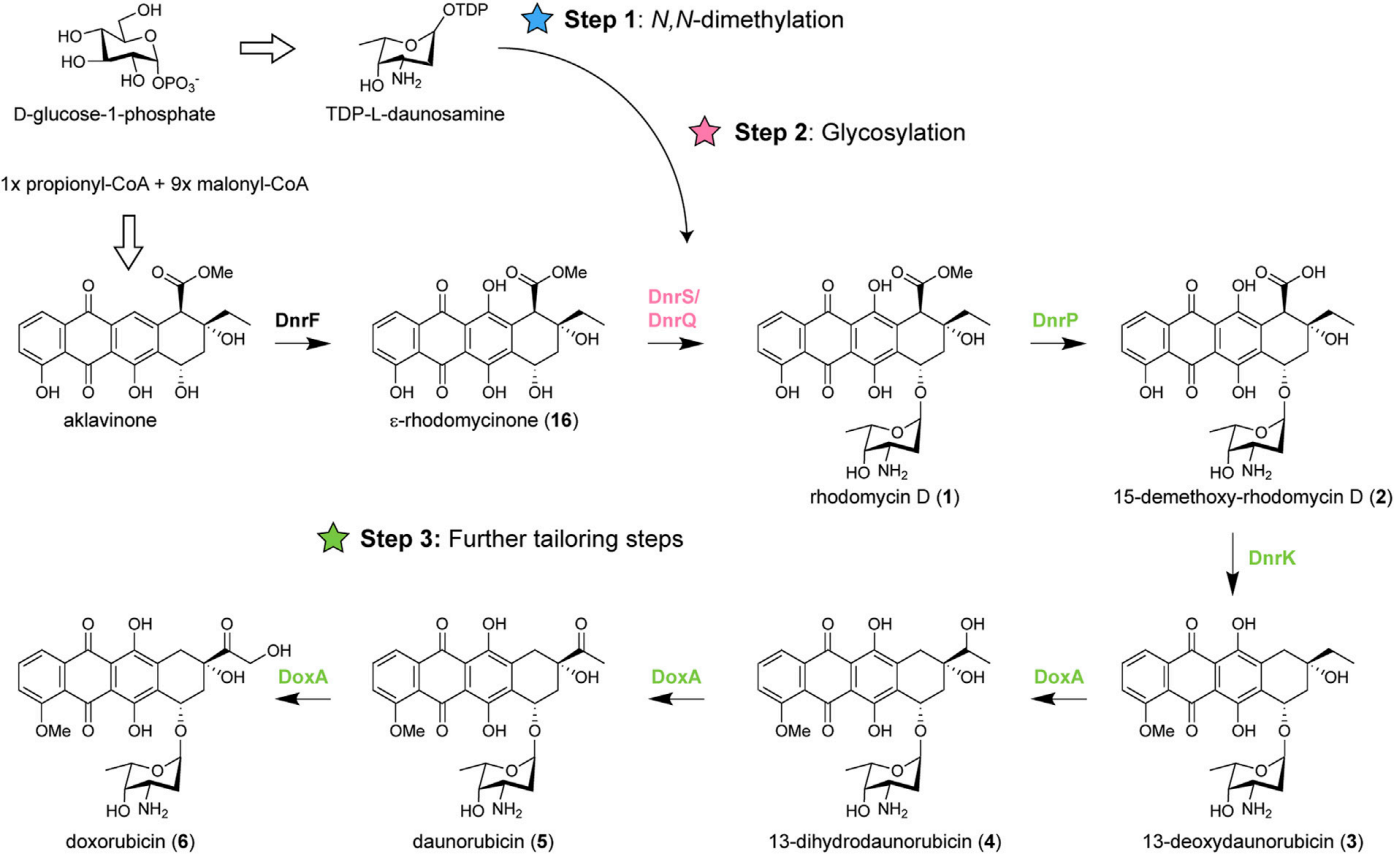
Biosynthetic pathway of doxorubicin and modifications required for biosynthesis of *N*,*N*-dimethylated daunorubicin and doxorubicin. Doxorubicin biosynthesis occurs in three stages: biosynthesis of TDP-l-daunosamine from D-glucose-1-phosphate, biosynthesis of ɛ-rhodomycinone (**16**) from one propionyl-CoA and nine malonyl-CoA units, followed by tailoring steps of the aglycone. For biosynthesis of *N,N*-dimethylated daunorubicin and doxorubicin, the pathway should be modified in three steps: 1) *N,N*-dimethylation of TDP- l-daunosamine to TDP-l-rhodosamine, 2) glycosylation of ɛ-rhodomycinone (**16**) with l-rhodosamine instead of l-daunosamine, and 3) the further tailoring steps should be performed when l-rhodosamine is attached.

Anthracycline biosynthetic pathways are generally divided into three stages: (amino) sugar biosynthesis, polyketide biosynthesis to generate the aglycone, followed by several tailoring steps of the aglycone including glycosylation (Metsä-Ketelä et al., 2008). Heterologous expression of genes from various anthracycline BGCs has previously been applied successfully as strategy for the biosynthesis of new anthracyclines (Brown et al., 2020; Hulst et al., 2022; Wang et al., 2022). For the biosynthesis of *N,N*-dimethylated daunorubicin and doxorubicin, the native doxorubicin biosynthetic pathway should be modified in three steps: (Step 1) *N,N*-dimethylation of TDP-l-daunosamine to TDP-l-rhodosamine, (Step 2) glycosylation of the anthracyclinone ε-rhodomycinone with l-rhodosamine instead of l-daunosamine, and (Step 3) further tailoring steps to achieve full conversion of the aglycone toward *N,N*-dimethyldoxorubicin (**12**) (Figure 2).

For Step 1, a methyltransferase could be introduced to catalyse the conversion of TDP-l-daunosamine to TDP-l-rhodosamine. The enzymatic synthesis of TDP-l-rhodosamine occurs via an *S*-adenosyl-l-methionine (SAM)-dependent *N*-methyltransferase (Siitonen et al., 2018). The aclarubicin biosynthetic pathway of *Streptomyces galilaeus* contains the AclP and AknX2 *N*-methyltransferases (Räty et al., 2000), which are both required for *N,N*-dimethylation of TDP-l-daunosamine (Han et al., 2011). For Step 2, a glycosyltransferase is required to attach rhodosamine to ε-rhodomycinone. Glycosyltransferases are well-described as promiscuous enzymes that can accept a wide range of substrates (Brown et al., 2020). The native glycosyltransferase DnrS may be able to glycosylate with rhodosamine but may also favour daunosamine. Alternatively, glycosyltransferases of the aclarubicin and rhodomycin biosynthetic pathway could be heterologously expressed. The final challenge (Step 3) involves further tailoring reactions catalysed by the 15-methyleserase DnrP, 4-*O*-methyltransferase DnrK, and cytochrome P450 monooxygenase DoxA (Dickens et al., 1997). An alternative for DnrP may be found in the rhodomycin pathway of *Streptomyces purpurascens*. RdmC is a homologue of DnrP that natively accepts ε-rhodomycin T (**7**) as substrate, which harbours the *N,N*-dimethylated amino sugar moiety (Grocholski et al., 2015). The following enzyme DnrK catalyses 4-*O*-methylation as well as moonlighting activity 10-decarboxylation, which is a unique feature among the characterised anthracycline methyltransferases (Jansson et al., 2004; Grocholski et al., 2015). The final enzyme DoxA is also unique to the doxorubicin pathway, where the conversion from daunorubicin (**5**) to doxorubicin (**6**) is notably inefficient (Walczak et al., 1999).

To achieve optimal production results, an industrial *S. peucetius* strain optimised for doxorubicin production was used as background strain. G001 is an industrial strain derived from *S. peucetius* by *N*-methyl-*N′*-nitro-*N*-nitrosoguanidine (NTG) mutagenesis (Lambert and Ylihonko, 2008). Compared to wild-type *S. peucetius*, the industrial strain produces more than hundred times more daunorubicin (**5**) and doxorubicin (**6**), which makes it an appropriate choice as parental strain for the engineering efforts. Here we present the outcomes of the genetic engineering strategies applied to biosynthetically produce *N*,*N*-dimethyldaunorubicin (**11**) and *N*,*N*-dimethyldoxorubicin (**12**). The results indicate that *N,N*-dimethyldaunorubicin (**11**) can be produced by combinatorial biosynthesis. However, further optimisation of DoxA is crucial to enhance the production titres of *N,N*-dimethyldaunorubicin (**11**) and achieve biosynthesis of *N,N*-dimethyldoxorubicin (**12**).

## 2 Materials and methods

### 2.1 Bacterial strains and growth conditions

The bacterial strains used in this work are listed in Table 1. *E. coli* strains JM109 (Yanisch-Perron et al., 1985), and ET12567/pUZ8002 (MacNeil et al., 1992) were used for routine cloning and for conjugation or isolation of non-methylated DNA, respectively. *E. coli* strains were cultivated at 37°C on Luria-Bertani (LB) agar plates or in LB medium supplemented with the appropriate antibiotics. All media and routine *Streptomyces* techniques have been described previously (Kieser et al., 2000). *S. peucetius* G001 (Lambert and Ylihonko, 2008) was used as parental strain. Soy flour mannitol (SFM) agar plates were used to grow *Streptomyces* for phenotypical characterisation. Tryptone soy broth (TSB) was used for liquid cultivation of *Streptomyces* strains. The growth media were supplemented with 20 μg mL^−1^ thiostrepton when required. Cultures were grown in a total volume of 20 mL of liquid medium in 100 mL Erlenmeyer flasks equipped with metal coils. Shake flaks were incubated in an orbital shaker with a 2-inch orbit at 200 rpm at 30°C. Due to poor sporulation of *S. peucetius* strains, mycelium stocks were prepared as an alternative to spore stocks. Strains were cultivated in TSB medium for 2 days, the biomass was washed with 10.3% (w/v) sucrose, resuspended in 20% (w/v) glycerol, and stored at −80°C.

**TABLE 1:** Strains used in this study.

Strain	Description	References
*Escherichia coli* JM109	For routine plasmid maintenance and cloning	Yanisch-Perron et al. (1985)
*Escherichia coli* ET12567/pUZ8002	Methylation-deficient strain for isolation of non-methylated DNA and conjugating plasmids into *Streptomyces*	MacNeil et al. (1992)
*Escherichia coli* TOP10	For protein expression	Invitrogen
*Streptomyces peucetius* var. *caesius* ATCC 27952	Derived from *Streptomyces peucetius* ATCC 29050, producer of daunorubicin and doxorubicin	Arcamone et al. (1969)
*Streptomyces peucetius* G001	Derived from ATCC 27952 by NTG mutagenesis; increased production of daunorubicin	Lambert and Ylihonko (2008)
MAG301	G001 + pRDS	This work
MAG302	G001 Δ*dnrS*	This work
MAG303	G001 Δ*dnrS* + pRDS	This work
MAG304	G001 Δ*dnrS* + pRDS + pGWS1432	This work
MAG305	G001 Δ*dnrS* + pGWS1433	This work
MAG306	G001 Δ*dnrS* + pRDS + pGWS1432 + pGWS1434	This work
MAG307	G001 Δ*dnrS* + pRDS + pGWS1432 + pGWS1435	This work
MAG308	G001 Δ*dnrS* + pRDS + pGWS1432 + pGWS1436	This work
MAG309	G001 Δ*dnrS* + pGWS1433 + pGWS1437	This work

### 2.2 Plasmids and strains generated in this study

All plasmids described in this work are listed in Supplementary Table S1 and primers in Supplementary Table S2. Plasmid maps were generated using SnapGene 6.0 (Supplementary Figure S1).

#### 2.2.1 Construct for gene disruption of *dnrS*

The strategy for creating deletion mutants is based on the unstable multicopy vector pWHM3 (Vara et al., 1989), as described previously (Świątek et al., 2012). Briefly, a knock-out construct was generated containing an apramycin resistance cassette that is flanked by the upstream and downstream region of the targeted gene. The about 1 kb upstream and downstream regions of *dnrS* (Table 2) were amplified from *S. peucetius* ATCC 27952 genomic DNA using primers MH301/MH302 and MH303/MH304. The DNA fragments were subsequently cloned into pWHM3 using EcoRI/HindIII. The apramycin resistance gene *aacC4* flanked by two *loxP* recognition sites was cloned in-between the flanking regions of *dnrS* using an engineered XbaI restriction site. The resulting knock-out construct, designated as pGWS1431 (Supplementary Figure S1), was verified using Sanger sequencing. Subsequently, the construct was introduced to G001 via protoplast transformation (Kieser et al., 2000). The desired double-crossover mutant was selected by resistance against apramycin (50 μg mL^−1^) and sensitivity to thiostrepton (20 μg mL^−1^). The presence of the *loxP* recognition sites allowed the efficient removal of the apramycin resistance cassette from the chromosome following the introduction of pUWLCRE that expresses the Cre recombinase (Fedoryshyn et al., 2008). The successful deletion of *dnrS* and removal of the apramycin resistance cassette was confirmed by gel electrophoresis of the PCR product of primers MH305/MH306. A distinct band was observed at the expected size of 489 (Supplementary Figure S2).

**TABLE 2:** Origin and function of genes and enzymes used in this study.

Gene	Enzyme	Size (nt/aa)	Origin	CDS	Catalytic function
*aknX2*	AknX2	717/238	*S. galilaeus*	CP966_RS29165	*N*-methyltransferase
*aclP*	AclP	732/243	*S. galilaeus*	CP966_RS29066	*N*-methyltransferase
*dnrS*	DnrS	1,296/431	*S. peucetius*	CGZ69_RS24500	Glycosyltransferase
*dnrQ*	DnrQ	1,317/438	*S. peucetius*	CGZ69_RS24505	Glycosyltransferase auxiliary protein
*aknS*	AknS	1,332/443	*S. galilaeus*	CP966_RS29040	Glycosyltransferase
*aknT*	AknT	1,332/443	*S. galilaeus*	CP966_RS29045	Glycosyltransferase auxiliary protein
*dnrP*	DnrP	885/294	*S. peucetius*	CGZ69_RS24510	15-Methylesterase
*rdmC*	RdmC	894/297	*S. purpurascens*	LYO46_16725	15-Methylesterase
*dnrK*	DnrK	1,071/356	*S. peucetius*	CGZ69_RS24515	4-*O*-methyltransferase (10-decarboxylase moonlighting activity)
*doxA*	DoxA	1,248/415	*S. peucetius*	CGZ69_RS24600	Cytochrome P450 monooxygenase
*doxA-1^a^*	DoxA-1	1,248/415	*S. peucetius*	CGZ69_RS24600	Cytochrome P450 monooxygenase
*doxA-2^a^*	DoxA-2	1,248/415	*S. bellus*	GCM10010244_ 64,990	Cytochrome P450 monooxygenase
*doxA-3^a^*	DoxA-3	1,248/415	*S. coeruleorubidus*	CP976_32970	Cytochrome P450 monooxygenase
*drrA*	DrrA	993/330	*S. peucetius*	CGZ69_RS24655	ABC-family transporter (ATP-binding subunit)
*drrB*	DrrB	852/283	*S. peucetius*	CGZ69_RS24650	ABC-family transporter (permease subunit)

^a^ Genes were codon-optimised based on the codon usage of *Streptomyces coelicolor*.

#### 2.2.2 Constructs for expression of biosynthetic genes

For the expression of biosynthetic genes, the integrative vector pSET152 (Bierman et al., 1992), the integrative vector pMS82 (Gregory et al., 2003), and the multicopy vector pWHM3-oriT (Wu et al., 2019) were employed. pSET152 and pMS82 integrate into the attachment sites within the *Streptomyces* genome for bacteriophages φC31 and φBT1, respectively. The vectors harbour the apramycin and hygromycin resistance cassettes, respectively. pWHM3-oriT is a derivative of pWHM3 (Vara et al., 1989) that harbours *oriT* to allow for its conjugative transfer and a thiostrepton resistance cassette.

To generate a construct for expression of *rdmC* from *S. purpurascens* ATCC 25489 (Table 2), the coding sequence (+0/+944, relative to the start codon of *rdmC*, amplified by primers MH307/MH308 from pBAD/HisB-rdmC (Grocholski et al., 2015)) under control of the constitutive ermE* promoter (Bibb et al., 1985) was cloned into pSET152 using EcoRI/XbaI (pGWS1432, Supplementary Figure S1).

The expression cassette of pRDS (Han et al., 2011) was cloned into pSET152 along with *rdmC* to generate a construct for expression of the sugar biosynthesis genes and tailoring genes for *N,N*-dimethyldoxorubicin (**12**) biosynthesis. In this construct, the capsid ribosomal binding site R15 from bacteriophage φC31 (Bai et al., 2015), the coding region of *rdmC*, and the L3S1P47 terminator (Chen et al., 2013) were cloned downstream the *aclP* gene in the pRDS expression cassette. The ribosomal binding site R15 was introduced to *rdmC* via primers MH309/MH310 (amplified from pGWS1432). The pUCK_L3S1P47 vector that harbours the L3S1P47 terminator was linearised by primers MH311/MH312. The two fragments were used to generate R15-*rdmC*-L3S1P47pUCK via Gibson assembly (Gibson et al., 2009). Subsequently, the R15-*rdmC*-L3S1P47 region was amplified by primers MH313/MH314. The coding sequence of *aclP* and its upstream region containing an EcoRI restriction site was amplified by primers MH315/HH316 from pRDS. The two fragments were cloned into EcoRI/BamHI linearised pSET152 vector via Gibson assembly (*aclP*-R15-*rdmC*-L3S1P47pSET152). The expression cassette of pRDS minus *aclP* was excised from the pRDS expression vector using EcoRI and cloned into the EcoRI linearised *aclP*-R15-*rdmC*-L3S1P47pSET152, resulting in pGWS1433-v1. The correct orientation of the fragment was verified by Sanger sequencing using the M13_R primer.

Illumina sequencing of pRDS indicated the presence of an unanticipated EcoRI site in the intergenic region between *aclP* and *aknX2*. Consequently, the 70 bp sequence between the EcoRI site within the coding region of *aknX2* and the EcoRI site in the intergenic region between *aclP* and *aknX2* is missing in pGWS1433-v1. The missing sequence was introduced to the construct via Gibson assembly. The *dnmU-dnmV-dnmJ-aknX2* region was amplified from pRDS by primers MH319/MH320, including the missing sequence at the end of *aknX2*. The *aclP-rdmC* region was amplified from pGWS1433-v1 by primers MH317/MH318. The two fragments were cloned into BamHI linearised pGWS1433-v1 via Gibson assembly, resulting in pGWS1433 (Supplementary Figure S1). The introduction of the 70 bp sequence was confirmed by Sanger sequencing using the M13_R primer.

Three constructs were designed for the expression of *doxA* from *S. peucetius*, and two heterologous *doxA* genes from *Streptomyces bellus* and *Streptomyces coeruleorubidus*, respectively (Table 2). The DoxA enzymes of both strains have 99.3% sequence identity with *S. peucetius* DoxA. The genes were codon optimised based on the native codon preference of *Streptomyces coelicolor* using GenSmart Design (GenScript Biotech Crop, NJ, USA). The coding sequences were flanked by the *gapdh* promoter P7 from *Tsukamurella paurometabola* (Bai et al., 2015) with the helicase ribosomal binding site R9 from bacteriophage φC31 (Bai et al., 2015) and the *aph* terminator (Pulido and Jiménez, 1987). The DNA fragments were synthesised by BaseGene (Leiden) and provided in pUC19 flanked by EcoRV sites. The fragments were cloned into pMS82 using EcoRV to generate pGWS1434 (*doxA*-1 of *S. peucetius*), pGWS1435 (*doxA*-2 of *S. bellus*), and pGWS1436 (*doxA*-3 of *S. coeruleorubidus*). The orientation of the fragments was determined by Sanger sequencing using the M13_R primer (Supplementary Figure S1).

To generate a construct for expression of *drrA* and *drrB* from *S. peucetius* ATCC 27952 (Table 2), the coding sequence of *drrAB* (+0/+1897 relative to the start codon of *drrA*, amplified by primers MH321/MH322) under control of the constitutive ermE* promoter was cloned into pWHM3-oriT using EcoRI/XbaI (pGWS1437, Supplementary Figure S1).

### 2.3 Metabolomics

#### 2.3.1 Metabolite extraction

*S. peucetius* strains were cultivated in E1 medium (Ylihonko et al., 1994), to which 5% (w/v) Diaion HP20 (Resindion SRL) was added prior to autoclaving. A 25 µL aliquot of mycelium stock was inoculated into 25 mL E1 medium in 100 mL Erlenmeyer flasks without metal coil. The cultures were incubated in a rotary shaker at 30°C for 4 days. Following fermentation, both resin and biomass were collected by vacuum filtration, washed with distilled water, and extracted three times with 25 mL acetone by overnight soaking. The acetone extracts were evaporated under a nitrogen flow at 40°C, and subsequently re-dissolved in 80% acetonitrile to obtain a final concentration of 1 mg mL^−1^ crude extract for LC-MS/MS analysis.

#### 2.3.2 LC-MS analysis

LC-MS/MS acquisition was performed using a Shimadzu Nexera X2 UHPLC system, with attached photodiode array detector (PDA), coupled to a Shimadzu 9030 QTOF mass spectrometer (MS), equipped with a standard electrospray ionisation (ESI) source unit, in which a calibrant delivery system (CDS) was installed. A total of 2 µL were injected into a Waters Acquity HSS C_18_ column (1.8 µm, 100 Å, 2.1 × 100 mm). The column was maintained at 30°C, and run at a flow rate of 0.5 mL min^−1^, using 0.1% formic acid in H_2_O as solvent A, and 0.1% formic acid in acetonitrile as solvent B. A gradient was employed for chromatographic separation starting at 15% B for 1 min, then 15%–60% B for 9 min, 60%–100% B for 1 min, and finally held at 100% B for 3 min. The column was re-equilibrated to 5% B for 3 min before the next run was started. The PDA acquisition was performed in the range 200–600 nm, at 4.2 Hz, with 1.2 nm slit width. The flow cell was maintained at 40°C.

All samples were analysed in positive polarity, using data dependent acquisition mode. In this regard, full scan MS spectra (*m/z* 100–2000, scan rate 20 Hz, ID disabled) were followed by three data dependent MS/MS spectra (*m/z* 100–2000, scan rate 20 Hz, ID disabled) for the three most intense ions per scan. The ions were selected when they reach an intensity threshold of 1,500, isolated at the tuning file Q1 resolution, fragmented using collision induced dissociation at fixed collision energy of 20 eV, and excluded for 0.01 s before being re-selected for fragmentation. The parameters used for the ESI source were: interface voltage 4 kV, interface temperature 300°C, nebulizing gas flow 3 L min^−1^, and drying gas flow 10 L min^−1^.

#### 2.3.3 Annotation of mass features in LC/MS data

Raw data obtained from LC-MS analysis were converted to mzXML centroid files using Shimadzu LabSolutions Postrun analysis. The files were imported into MZmine 2.53 (Pluskal et al., 2010) for data processing. Extracted ion chromatograms were generated with an *m/z* tolerance set to 0.002 m*/z* or 10.0 ppm.

For statistical analysis, LC-MS data were processed as described previously (van Bergeijk et al., 2022). Briefly, mass ion peaks were detected (positive polarity, mass detector: centroid) and their chromatograms were built using ADAP chromatogram builder (Myers et al., 2017) (minimum group size in number of scans: 10; group intensity threshold: 200). The detected peaks were smoothed (filter width: 9), and the chromatograms were deconvoluted (algorithm: local minimum search; chromatographic threshold: 85%; search minimum in RT range: 0.05; minimum relative height: 1%; minimum ratio of peak top/edge: 2; peak duration: 0.03–2.00 min). The detected peaks were deisotoped (monotonic shape; maximum charge: 2; representative isotope: most intense). The peak list was exported as a comma-separated file. Data of three independent replicates were used to calculate the change in the mass peak areas of the different metabolites across the different tested strains.

LC-MS data used to identify compounds **1**–**16** in the extracts of the tested strains is provided in Supplementary Table S3 and Supplementary Figures S6–S18. Compounds **3** (van Gelder et al., 2023), **5** (Sanofi BV), **6** (Accord Healthcare Limited), **9** (van Gelder et al., 2023), **11** (van Gelder et al., 2023) and **12** (Qiao et al., 2020) were identified by matching the retention time, HRMS and HRMS/MS spectra to reference compounds. Compounds **4**, **7**, **10**, **13**–**16** were annotated based on their calculated exact mass and expected MS/MS spectra. Compounds **1**, **2**, **8** and **12** could not be detected in the crude extracts of the engineered strains.

### 2.4 Proteomics

MAG304 was cultivated in E1 medium. Biomass was harvested after 2, 3 or 4 days of incubation (*n* = 3), snap-frozen in liquid nitrogen and stored at −80°C until analysis. The frozen biomass was lysed using a Bioruptor Plus (Diagenode SA) and proteins were extracted using lysis buffer [4% SDS, 100 mM Tris-HCl (pH 7.6), 50 mM EDTA]. Sample preparation for LC-MS/MS measurement was performed as described previously (Zhang et al., 2020). Briefly, total protein was precipitated using the chloroform-methanol method (Wessel and Flügge, 1984) and dissolved in 0.1% RapiGest SF surfactant (Waters Crop.) at 95 °C. The protein concentration was determined using the BCA method. Protein samples were reduced by adding 5 mM dithiothreitol (DTT) and incubated in the dark at 60°C for 30 min, followed by thiol group protection using 21.6 mM iodoacetamide and incubation in the dark at room temperature for 30 min. Subsequently, 0.1 µg of trypsin (recombinant, proteomics grade, Roche) per 10 µg of protein was added, and samples were digested overnight at 37°C. After digestion, trifluoroacetic acid was added to a concentration of 0.5%. The samples were incubated at 37°C for 30 min, followed by centrifugation to degrade and remove the RapiGest SF. The resulting peptide solution, containing 6 µg of peptides, was cleaned and desalted using StageTips (Rappsilber et al., 2007). Briefly, 6 µg of peptides was loaded on a conditioned StageTip with two 1 mm diameter C_18_ disks (Empore, product number 2215), washed twice using a 0.5% formic acid solution, and eluted with elution solution (80% acetonitrile and 0.5% formic acid). Acetonitrile was evaporated using a SpeedVac. The final peptide concentration was adjusted to 40 ng μL^−1^ using sample solution (3% acetonitrile and 0.5% formic acid) for analysis. Quantitative proteomics was performed as described previously (Zhang et al., 2020). Briefly, the desalted peptide solution was separated using an UltiMate 3,000 RSLCnano system (Thermo Scientific) set in a trap-elute configuration, coupled with a QExactive HF mass spectrometer (Thermo Scientific). The liquid chromatography system used a Waters nanoEase M/Z Symmetry C_18_ trap column (5 μm, 100 Å, 180 μm × 20 mm) for peptide loading and retention, and a Waters nanoEase M/Z HSS T3 C_18_ analytical column (1.8 µm, 100 Å, 75 μm × 250 mm) for peptide separation. The mass spectrometer was operated in positive mode with data-dependent acquisition. Raw LC-MS/MS files were analysed using MaxQuant software v2.2.0.0 (Cox and Mann, 2008) using the label-free quantification (LFQ) method.

### 2.5 Bioinformatics

The BGCs of doxorubicin/daunorubicin, aclarubicin and rhodomycin were visualised using clinker (Gilchrist and Chooi, 2021). DoxA homologs were identified using NCBI BLASTP search (http://blast.ncbi.nlm.nih.gov). Alignment of the obtained protein sequences was performed using Cluster Omega 1.2.4 (Madeira et al., 2022).

### 2.6 Enzymatic assays

Enzymatic activity assays were conducted as described elsewhere in detail (Koroleva et al., 2024). The DoxA, DnrV, FDX4 and SFR proteins were produced as N-terminally 6×His-tagged recombinant proteins in *E. coli* TOP10 and purified by affinity chromatography using TALON Superflow resin (GE Healthcare). Proteins were concentrated using Amicon Ultra-4 10K centrifugal filters (Merck Millipore) and stored at −20°C in 40% glycerol. The proteins were analysed for purity and molecular weight using SDS-PAGE. Enzymatic activity measurements were carried out at room temperature overnight. Then, reactions were extracted with 4:1 mixture of chloroform and methanol. The extracts were evaporated using a vacuum concentrator and dissolved in methanol for HPLC analysis. HPLC analysis was performed using a Shimadzu Nexera X3 system with a PDA detector and a Phenomenex Kinetex C_18_ column (2.6 µM, 100 Å, 4.6 × 100 mm). The column was run at a flow rate of 0.5 mL min^−1^, using 0.1% formic acid, 15% acetonitrile and 85% H_2_O as solvent A, and 100% acetonitrile as solvent B. A gradient was employed starting at 100% A for 2 min, then 0%–60% B for 18 min, 100% B for 4 min, and finally 100% A for 5 min. The absorbance of the samples was recorded at 490 nm. The reaction products were identified by comparison to reference compounds for **3**–**6**, **9**, **11** and **12**. For enzymatic assays with compound **9** and **11**, high resolution electrospray ionization mass spectra were recorded on a Waters Acquity RDa detector using a Waters XBridge BEH C_18_ column (5 μm, 130 Å, 4.6 × 30 mm). The column was run at a flow rate of 0.8 mL min^−1^, using 0.1% formic acid in H_2_O as solvent A, and 0.1% formic acid in acetonitrile as solvent B. A gradient was employed starting at 2%–100% B for 132 s, then 100% B for 18 s, 100%–2% B for 18 s min, and finally 2% B for 12 s. The reaction products were identified by comparison to reference compounds for **11** (Supplementary Figure S19) and **12** (Supplementary Figure S20). Compound **10** was annotated based on calculated exact mass (Supplementary Figure S21).

### 2.7 Microbial inhibition assays

To investigate the resistance of *S. peucetius* to anthracyclines, 5 µL of mycelium stock was spotted at a concentration of 1.0.10^4^ colony forming units (CFU) per spot on SFM agar plates supplemented with increasing concentrations of 13-deoxydaunorubicin **3**) (van Gelder et al., 2023), doxorubicin **6**) (Accord Healthcare Limited), *N,N*-dimethyl-13-deoxydaunorubicin **9**) (van Gelder et al., 2023) or *N,N*-dimethyldoxorubicin (**12**) (Qiao et al., 2020). After 3 or 4 days of incubation at 30 °C, growth was examined visually.

## 3 Results

### 3.1 Attachment of l-rhodosamine to ɛ-rhodomycinone via heterologous expression of aclarubicin sugar *N*-methyltransferases and glycosyltransferases

**FIGURE 3:**
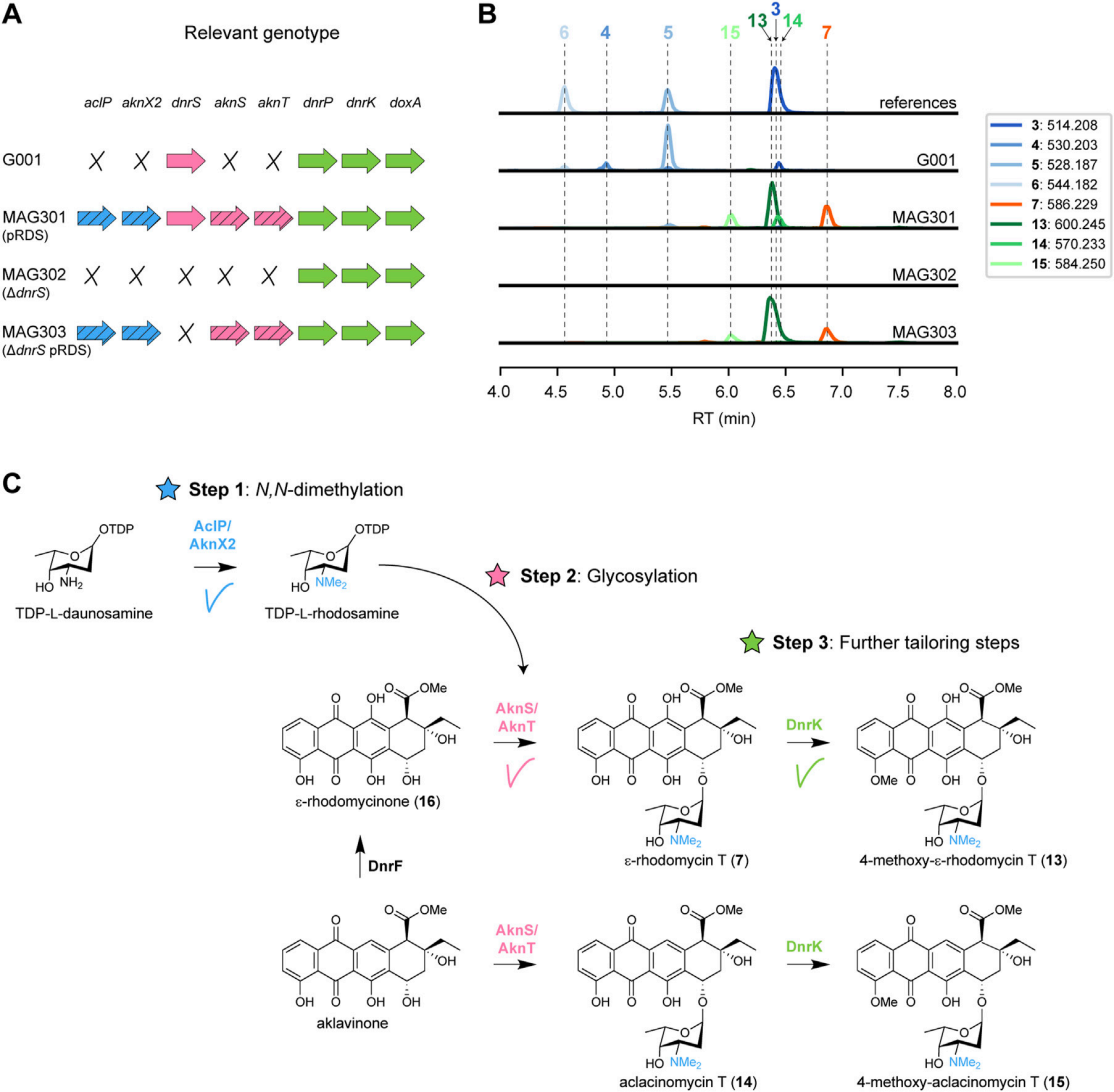
Expression of aclarubicin methyltransferases and glycosyltransferases genes in G001 results in the attachment of l-rhodosamine to ɛ-rhodomycinone **(A)** Schematic representation of the relevant genotype of the strains used in this experiment, with heterologous genes indicated by a diagonal striped pattern. **(B)** LC-MS analysis of crude extracts of G001, MAG301, MAG302 and MAG303 cultivated in E1 medium. Extracted ion chromatograms showing the mass peaks [M + H]^+^ of compounds **3**–**7** and **13**–**15**. **(C)** Schematic representation of the engineered doxorubicin pathway. Introduction of the *N*-methyltransferases (*aclP/aknX2*) and glycosyl transferases (*aknS*/*aknT*) genes from the aclarubicin BGC resulted in incorporation of l-rhodosamine onto ɛ-rhodomycinone (**16**), forming ɛ-rhodomycin T (**7**). ɛ-Rhodomycin T (**7**) was converted to 4-methoxy-ɛ-rhodomycin T (**13**) by DnrK. Additionally, a minor mass peak of daunorubicin (**5**) was detected in MAG301, which was completely abolished in MAG303 where the native glycosyltransferase gene *dnrS* was deleted.

The first challenge in the biosynthetic production of *N,N*-dimethyldaunorubicin (**11**) and *N,N*-dimethyldoxorubicin (**12**) is to provide the *N,N*-dimethylated amino sugar l-rhodosamine that cannot be naturally biosynthesised by *S. peucetius*. l-Rhodosamine occurs in various natural anthracyclines, such as aclarubicin and rhodomycin B (Figure 1A). The aclarubicin biosynthetic pathway of *S. galilaeus* features two *N-*methyltransferases, AclP and AknX2, which can catalyse the conversion of the activated amino sugar TDP-l-daunosamine to TDP-l-rhodosamine (Step 1, Figure 3C). Additionally, the aclarubicin biosynthetic pathway contains the glycosyltransferase/auxiliary protein pair AknS and AknT, which normally catalyse the glycosylation of aklavinone with rhodosamine to yield aclacinomycin T (**14**). However, the doxorubicin biosynthetic pathway differs from the aclarubicin pathway by the presence of DnrF, which catalyses the 11-hydroxylation of aklavinone to ɛ-rhodomycinone (**16**) before glycosylation (Figure 3C). If AknS/AknT would be able to glycosylate ɛ-rhodomycinone (**16**) with rhodosamine, it would result in the production of ɛ-rhodomycin T **7**) (Step 2).

The construct pRDS (Han et al., 2011) harbours the methyltransferase genes *aclP* and *aknX2* and the glycosyltransferase genes *aknS* and *aknT* from *S. galilaeus,* and genes for the biosynthesis of TDP-l-rhodosamine from *S. peucetius* and *Streptomyces venezuelae*. This construct is a derivative of pWHM3 (Vara et al., 1989), an unstable multicopy vector that harbours a thiostrepton cassette (Supplementary Figure S1). pRDS was introduced into G001 via protoplast transformation, resulting in strain MAG301. The recombinant strain harbours all the genes for the enzymes required for both Step 1 and Step 2 (Figure 3A). To evaluate the effect of introducing pRDS on the metabolite profile, both G001 and MAG301 were cultivated in E1 medium (Ylihonko et al., 1994) with added HP20 resin. The resin binds the anthracyclines, thereby preventing product inhibition and toxicity. After 4 days of incubation at 30 °C, metabolites were extracted using acetone, dried, re-dissolved in 80% acetonitrile and analysed using liquid chromatography-mass spectrometry (LC-MS). The LC-MS data were processed using MZmine, resulting in a list containing all the mass features and their peak areas detected in each crude extract. In cases where reference compounds were unavailable, annotation was based on the predicted *m/z* values of the [M + H]^+^ adduct ions and expected fragmentation patterns (Supplementary Table S3).

The LC-MS chromatograms of G001 and MAG301 (G001 pRDS) exhibited distinctly different mass peaks (Figure 3B). The main peak observed in the chromatogram of G001 corresponded to daunorubicin **5**), and a smaller peak was annotated as doxorubicin **6**). Furthermore, minor peaks were annotated as the precursors 13-deoxydaunorubicin **3**) and 13-dihydrodaunorubicin **4**), respectively (Figure 3B). The introduction of pRDS to G001 (MAG301) resulted in a shift in the metabolite profile (Figure 3B). The main mass peaks detected in the LC-MS chromatogram of MAG301 were annotated as to ɛ-rhodomycin T **7**) and 4-methoxy-ɛ-rhodomycin T (**13**). These results indicate that ɛ-rhodomycinone (**16**) was successfully glycosylated with rhodosamine, yielding ɛ-rhodomycin T **7**) (Figure 3C). The main peak corresponding to 4-methoxy-ɛ-rhodomycin T (**13**) indicated that 4-*O*-methylation activity was successful, but 10-decarboxylation and the final hydroxylation steps were unsuccessful. In the native doxorubicin pathway, DnrP catalyses 15-methylesterase activity of rhodomycin D **1**), and subsequently DnrK catalyses 10-decarboxylation. The accumulation of 4-methoxy-ɛ-rhodomycin T (**13**) in the engineered pathway suggested that DnrP cannot catalyse 15-methylesterase activity of ɛ-rhodomycin T **7**) or 4-methoxy-ɛ-rhodomycin T (**13**).

Furthermore, minor peaks were observed in the chromatogram of MAG301, which were annotated as aclacinomycin T (**14**) and 4-methoxy-aclacinomycin T (**15**), respectively (Figure 3C). The presence of these compounds suggests that 11-hydroxylation of aklavinone to ɛ-rhodomycinone (**16**) by DnrF was not complete. All in all, the expression of sugar *N*-methyltransferases and glycosyltransferases of the aclarubicin biosynthetic pathway resulted in the incorporation of rhodosamine onto ɛ-rhodomycinone (**16**) (Figure 3C). However, optimisation of the further tailoring reactions toward *N,N*-dimethyldoxorubicin (**12**) are required.

### 3.2 Deletion of the native glycosyltransferase gene *dnrS*

The chromatogram of MAG301 also contained a minor mass peak that corresponds to daunorubicin **5**), indicating partial glycosylation of ɛ-rhodomycinone (**16**) with daunosamine rather than with rhodosamine (Figure 3B). The native doxorubicin glycosyltransferase DnrS may prefer TDP-l-daunosamine over TDP-l-rhodosamine, which could explain the observed by-products. To address this hypothesis, the native glycosyltransferase gene *dnrS* within the doxorubicin BGC was deleted (Figure 3A).

A deletion mutant of *dnrS* was created using a method published previously (Świątek et al., 2012), which is based on the unstable multicopy plasmid pWHM3 (Vara et al., 1989). A knock-out construct was generated that harbours the about 1 kb regions upstream and downstream of *dnrS* interspaced by the apramycin resistance gene *aacC4* flanked by *loxP* recognition sites (pGWS1431, Supplementary Figure S1). The knock-out construct was introduced to G001 via protoplast transformation. The presence of the *loxP* recognition sites allowed the efficient removal of the apramycin resistance cassette by introduction of the pUWLCRE construct for expression of the Cre recombinase (Fedoryshyn et al., 2008). Consequently, we obtained a mutant where the entire coding region of *dnrS* was deleted, which is designated MAG302 (Figure 3A).

MAG302 (G001 Δ*dnrS*) was cultivated in E1 medium, and the metabolite profile was analysed in a similar manner as described above. As expected, no glycosylated anthracyclinones could be detected in the LC-MS chromatogram of MAG302 (Figure 3B). The main peak in the chromatogram of MAG302 corresponded to ɛ-rhodomycinone (**16**) (Supplementary Figure S3). Subsequently, pRDS was introduced into MAG302 via protoplast transformation to generate MAG303 (Figure 3A). In contrast to MAG301, MAG303 harbours only the aclarubicin glycosyltransferases, but not the doxorubicin glycosyltransferase. MAG303 (G001 Δ*dnrS* pRDS) was cultivated in E1 medium, and the metabolite profile was analysed in a similar manner as described above. Notably, the production of daunorubicin **5**) was completely abolished in MAG303 (Figure 3B). When solely aclarubicin glycosyltransferases are present, ɛ-rhodomycinone (**16**) undergoes glycosylation exclusively with rhodosamine instead of daunosamine. These results indicate that the deletion of *dnrS* successfully directed the pathway toward glycosylation with rhodosamine.

### 3.3 Heterologous expression of *rdmC* enables *N*,*N*-dimethyldaunorubicin biosynthesis

After successful sugar *N*-methylation (Step 1) and glycosylation (Step 2), the next bottleneck in the *N,N*-dimethyldoxorubicin biosynthetic pathway lies in the downstream tailoring reactions (Step 3). The production of 4-methoxy-ɛ-rhodomycin T (**13**) indicated that DnrP could not catalyse 15-methylesterase activity of *N,N*-dimethylated substrates. Consequently, we searched for an alternative enzyme with 15-methylesterase activity of ɛ-rhodomycin T **7**).

**FIGURE 4:**
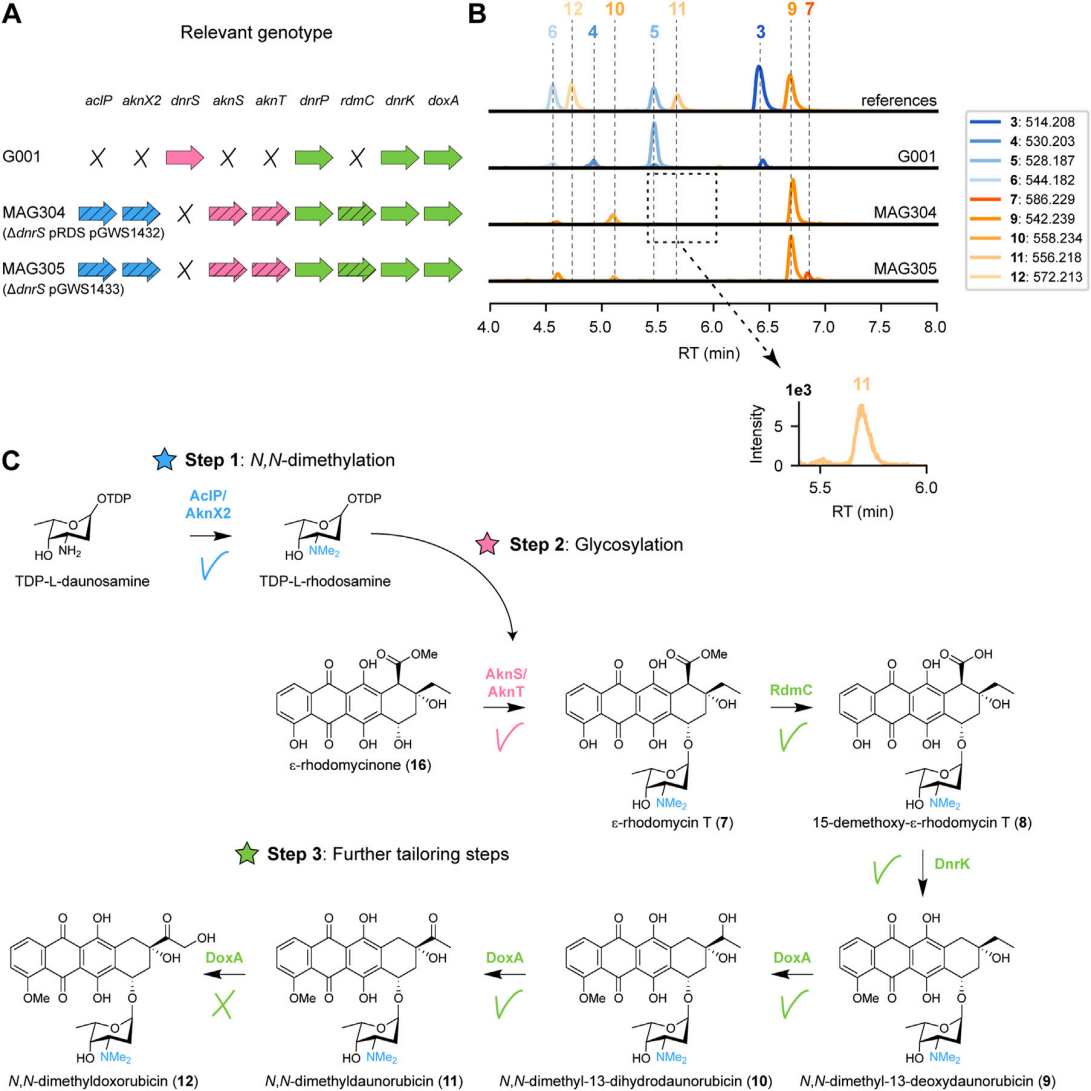
Expression of rhodomycin methylesterase gene in G001 results in the production of *N*,*N*-dimethyldaunorubicin. **(A)** Schematic representation of the relevant genotype of the strains used in this experiment, with heterologous genes indicated by a diagonal striped pattern. **(B)** LC-MS analysis of crude extracts of G001, MAG304 and MAG305 cultivated in E1 medium. Extracted ion chromatograms showing the mass peaks [M + H]^+^ of compounds **3**–**7** and **9**–**12**. **(C)** Schematic representation of the proposed biosynthetic pathway for *N,N*-dimethyldoxorubicin (**12**). Introduction of the 15-methylesterase gene *rdmC* from the rhodomycin BGC resulted in the production of *N,N*-dimethyl-13-deoxydaunorubicin (**9**) and *N,N*-dimethyl-13-dihydrodaunorubicin (**10**). A minor peak was detected for *N,N*-dimethyldaunorubicin (**11**), but *N,N*-dimethyldoxorubicin (**12**) could not be detected.

In *S. purpurascens*, the rhodomycin biosynthetic pathway harbours the enzyme RdmC, a homologue of DnrP, which accepts ɛ-rhodomycin T **7**) as substrate (Grocholski et al., 2015). The coding region of *rdmC* was positioned behind the constitutive ermE* promoter (Bibb et al., 1985), and cloned into the integrative vector pSET152 (Bierman et al., 1992), resulting in pGWS1432 (Supplementary Figure S1). The construct was introduced to MAG303 via conjugation, resulting in strain MAG304. The resulting strain harbours the methyltransferases and glycosyltransferases genes from the aclarubicin BGC (on pRDS), the methylesterase gene *rdmC* from the rhodomycin BGC (on pGWS1432), and a deletion of the native glycosyltransferase (*dnrS*) (Figure 4A). MAG304 (G001 Δ*dnrS* pRDS + *rdmC*) was cultivated in E1 medium, and the metabolite profile was analysed in a similar manner as described above. The main peak observed in the chromatogram of MAG304 was annotated as *N,N*-dimethyl-13-deoxydaunorubicin **9**), and a smaller peak as *N,N*-dimethyl-13-dihydrodaunorubicin (**10**). Remarkably, a minor peak was annotated as *N,N*-dimethyldaunorubicin (**11**), which is one of the targeted products (Figure 3B). Additionally, the production of ɛ-rhodomycin T **7**), 4-methoxy-ɛ-rhodomycin T (**13**), aclacinomycin T (**14**) and 4-methoxy-aclacinomycin T (**15**) was abolished (Figure 4B). The metabolite profile of MAG304 indicated successful replacement of DnrP by RdmC. The 15-methylesterase activity of RdmC is essential for the 10-decarboxylation moonlighting activity of DnrK, thus enabling the production of *N,N*-dimethyl-13-deoxydaunorubicin **9**). The final three steps from *N,N*-dimethyl-13-deoxydaunorubicin **9**) to *N,N*-dimethyldoxorubicin (**12**) are catalysed by the cytochrome P450 monooxygenase DoxA (Figure 4C). For G001, the main peak in the chromatogram corresponds to daunorubicin **5**), which indicates that the final 14-hydroxylation step toward doxorubicin **6**) is inefficient, while the two 13-hydroxylation steps catalysed by DoxA exhibit high efficiency. In contrast, for MAG304, the main peak corresponds to *N,N*-dimethyl-13-deoxydaunorubicin **9**), indicating that all steps catalysed by DoxA are inefficient when the compounds are *N,N*-dimethylated.

Taken together, introduction of *rdmC* into the engineered G001 strain proved to be a successful strategy to achieve 10-decarboxylation of ɛ-rhodomycin T **7**). Combined activity of RdmC and DnrK resulted in the production of *N,N*-dimethyldaunorubicin (**11**). The accumulation of the precursor *N,N*-dimethyl-13-deoxydaunorubicin **9**) indicates that the next bottleneck in the engineered pathway is the final enzyme DoxA.

To enable further genetic engineering, the genes located on pRDS and pGWS1432 were combined on a single integrative vector. For this, the coding region of *rdmC* with an engineered R15 ribosomal binding site (Bai et al., 2015) and L3S1P47 terminator (Chen et al., 2013) was amplified by PCR. The *aclP* coding region was amplified by PCR from pRDS. The two fragments were introduced into EcoRI-digested pSET152. The entire DNA fragment containing *aknT*-*aknS*-*desIII*-*desIV*-*dpsG*-*dpsH*-*dnmT*-*dnmZ*-*dnmU* was excised from pRDS and cloned into *aclP*-*rdmC*:pSET152 to generate pGWS1433-v1. Illumina sequencing of pRDS indicated the presence of an EcoRI site in the intergenic region between *aclP* and *aknX2* (Supplementary Figure S1). Consequently, our cloning strategy resulting in the loss of a fragment of 70 bp from the construct, which we then re-introduced. For this, the *dnmU-dnmV-dnmJ-aknX2* region was amplified from pRDS, including the missing sequence at the end of *aknX2*. The *aclP-rdmC* region was amplified by PCR from pGWS1433-v1. The two fragments were cloned into BamHI-linearised pGWS1433-v1 via Gibson assembly (Gibson et al., 2009), resulting in pGWS1433 (Supplementary Figure S1). The correct sequence of the whole construct was confirmed by Sanger sequencing. The resulting construct was introduced to MAG302 (G001 Δ*dnrS*) via conjugation to generate MAG305 (Figure 4A). MAG304 and MAG305 harbour the same heterologous genes. However, in the case of MAG305, all the heterologous genes are located on pSET152, whereas in the case of MAG304 all heterologous genes are located on pRDS, except for *rdmC* which is located on pSET152. MAG305 was cultivated in E1 medium, and the metabolite profile was analysed and annotated in a similar manner as described above. Similar as for MAG304, the main peak in the chromatogram of MAG305 corresponded to *N,N*-dimethyl-13-deoxydaunorubicin **9**), while *N,N*-dimethyldoxorubicin (**12**) could not be detected (Figure 4B), indicating that this strain could be use for further engineering.

### 3.4 DoxA is the bottleneck for biosynthesis of *N,N*-dimethyldoxorubicin

Heterologous expression of enzymes from the aclarubicin and rhodomycin pathways to G001 resulted in the biosynthesis of *N,N*-dimethyldaunorubicin (**11**). The results indicated that the bottleneck in the engineered biosynthetic pathway are the final three tailoring steps catalysed by DoxA. It is worth noting that the final 14-hydroxylation step catalysed by DoxA is notably inefficient in the doxorubicin biosynthetic pathway (Malla et al., 2010). In fact, enzyme kinetic experiments revealed that the catalytic constant V_max_ is 520-fold lower for the conversion of daunorubicin **5**) to doxorubicin **6**) compared to the preceding step (Walczak et al., 1999).

#### 3.4.1 Abundance of DoxA

**FIGURE 5:**
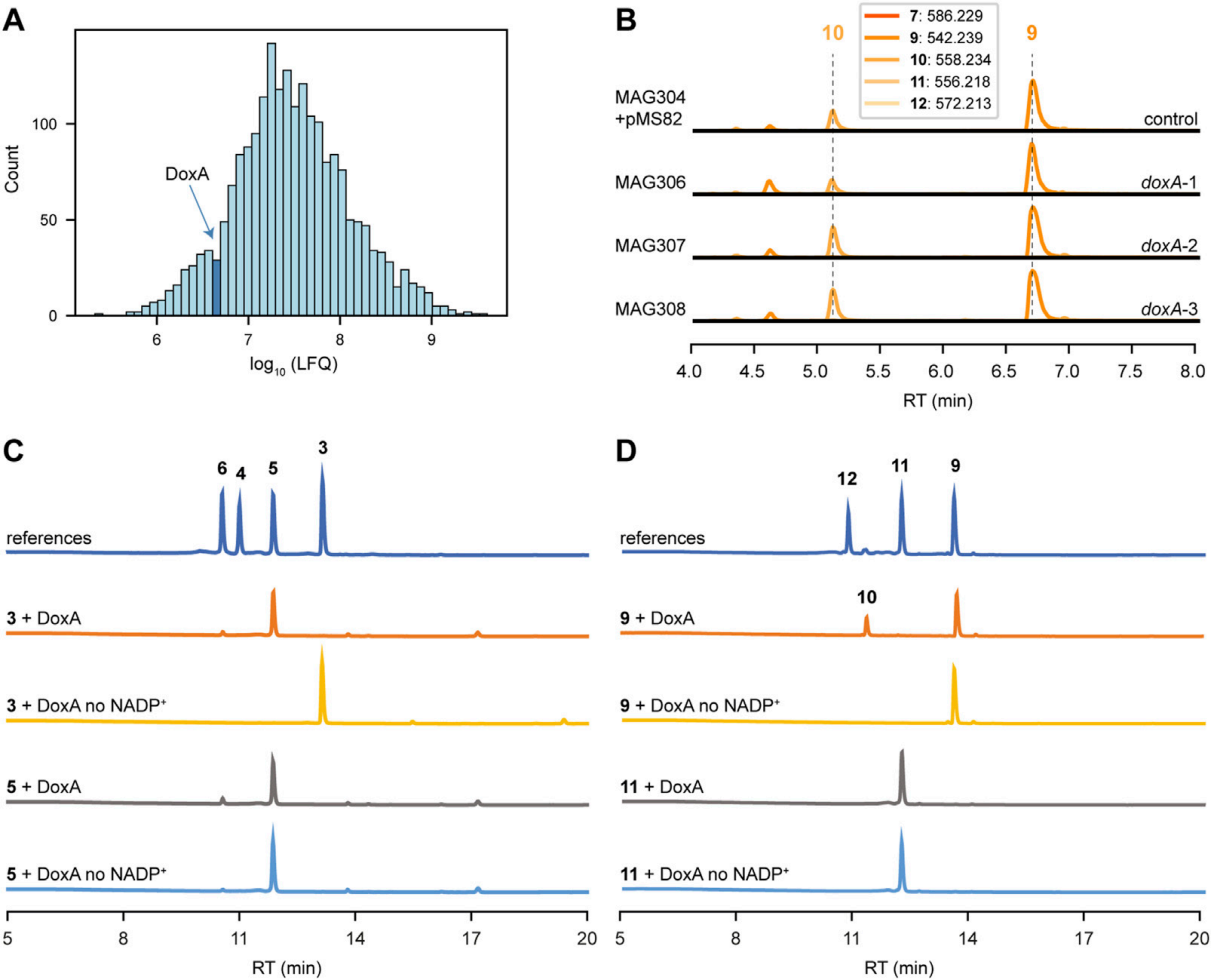
DoxA is the bottleneck for biosynthesis of *N*,*N*-dimethyldoxorubicin. **(A)** MS-based quantitative proteomics analysis of MAG304 cultivated in E1 medium for 3 days. Histogram showing distribution of the relative intensity level (log_10_ LFQ) of all detected proteins (*n* = 3). The bar that includes the abundance of DoxA is highlighted. **(B)** LC-MS analysis of crude extracts of MAG304 pMS82, MAG306 (*doxA*-1)*,* MAG307 (*doxA*-2) and MAG308 (*doxA*-3) cultivated in E1 medium. Extracted ion chromatograms showing the mass peaks [M + H]^+^ of compounds **7**–**12**. For all strains, the main peak corresponded to *N,N*-dimethyl-13-deoxydaunorubicin (**9**), and *N,N*-dimethyldoxorubicin (**12**) could not be detected. **(C)** HPLC analysis of DoxA enzymatic assays. UV-Vis chromatogram traces were recorded at 490 nm. A reaction mixture without the addition of NADP^+^ was used as the negative control. The activity of DoxA with the natural substrates 13-deoxydaunorubicin (**3**) and daunorubicin (**5**). **(D)** The activity of DoxA with *N*,*N*-dimethyl substrates *N*,*N*-dimethyl-13-deoxydaunorubicin (**9**) and *N*,*N*-dimethyldaunorubicin (**11**).

Quantitative proteomics was performed to analyse the abundance of DoxA in the engineered strain MAG304. The strain was cultivated in E1 medium, and biomass was collected after 2, 3 and 4 days (*n* = 3). In all samples, at least one peptide was detected that could be connected to DoxA. The abundance of all proteins was quantified via LFQ analysis, which provides a normalised concentration based on the presence of at least two peptide sequences for each protein. In 4 days-old-cultures only one peptide could be connected to DoxA, which is below the threshold. In the samples from 2 to 3 days-old-cultures, the abundance of DoxA was higher than 30% and 29% of other detected proteins (*n* = 3), respectively (Figure 5A). The results suggest that the abundance of DoxA is not likely the limiting factor for its activity (see Discussion).

#### 3.4.2 Heterologous expression of *doxA*

DoxA catalyses multi-step oxidation reactions on different carbon atoms (Figure 2). The oxidation reactions catalysed by DoxA are a unique feature of the doxorubicin pathway that is not found for other anthracyclines. To our knowledge, no DoxA homologue is known to accept *N,N*-dimethylated substrates. However, it may be possible to find an alternative DoxA enzyme with improved activity. All known members of the DoxA family originate from daunorubicin or doxorubicin producers, namely, *S. peucetius* ATCC 27952, *S. peucetius* ATCC 29050, *Streptomyces* sp. C5 and *S. coeruleorubidus*. A BLASTP search with *S. peucetius* DoxA also indicated that the genome of *S. bellus* also encodes a closely related DoxA enzyme (Supplementary Figure S4). The DoxA enzymes of *S. peucetius* ATCC 27952 and ATCC 29050 are identical. The DoxA enzymes of *Streptomyces* sp. C5, *S. coeruleorubidus* and *S. bellus* share 95.0%, 99.3% and 99.3% identity with *S. peucetius* DoxA, respectively. We decided to express the *S. bellus* and *S. coeruleorubidus doxA* genes each individually in the engineered strain MAG304.

The coding sequences of *S. peucetius*, *S. bellus* and *S. coeruleorubidus doxA* were codon-optimised based on the native codon preference of *S. coelicolor*. To optimise expression of the *doxA* genes, the respective coding sequences were positioned behind the strong *gapdh* promoter P7 (Bai et al., 2015) and the helicase ribosomal binding site R9 from bacteriophage φC31 (Bai et al., 2015), while the *aph* terminator (Pulido and Jiménez, 1987) was positioned behind the genes. The DNA fragments were synthesised and cloned into pMS82 (Gregory et al., 2003) using EcoRV to generate pGWS1434 (*doxA*-1 of *S. peucetius*), pGWS1435 (*doxA*-2 of *S. bellus*), and pGWS1436 (*doxA*-3 of *S. coeruleorubidus*). The constructs (Supplementary Figure S1) were conjugated into MAG304 to generate MAG306 (*doxA*-1), MAG307 (*doxA*-2) and MAG308 (*doxA*-3). The strains were cultivated in E1 medium, and the metabolite profile was analysed and annotated in a similar manner as described above. For all strains, the main peak in the chromatograms corresponded to *N,N*-dimethyl-13-deoxydaunorubicin **9**), while *N,N*-dimethyldoxorubicin (**12**) could not be detected (Figure 5B). The expression of heterologous and codon-optimised *doxA* genes did not affect the metabolite profile of the engineered strain. The results suggest that the alternative DoxA enzymes have similar activity as *S. peucetius* DoxA.

#### 3.4.3 Enzymatic assays DoxA

To evaluate the ability of DoxA to catalyse oxidation reactions on different substrates, *in vitro* enzyme activity was tested using the natural substrates 13-deoxydaunorubicin **3**) and daunorubicin **5**), and their *N,N*-dimethylated derivatives. HPLC analysis of the reaction products revealed that DoxA effectively converted 13-deoxydaunorubicin **3**) to both daunorubicin **5**) and doxorubicin **6**), with daunorubicin **5**) being the predominant product. The reaction with daunorubicin **5**) only resulted in a minor conversion to doxorubicin **6**) (Figure 5C). These results are consistent with *in vivo* findings, where cultivation of G001 primarily yields daunorubicin **5**) with minor amounts of doxorubicin **6**).

In contrast, when *N,N*-dimethyl-13-deoxydaunorubicin **9**) was utilised as substrate, DoxA exclusively catalysed the reaction toward *N,N*-dimethyl-13-dihydrodaunorubicin (**10**), and the conversion was found to be incomplete (Figure 5D). DoxA did not exhibit catalytic activity toward *N,N*-dimethyldaunorubicin (**11**) (Figure 5D). LC-MS analysis of the reaction product confirmed that no *N,N*-dimethyldoxorubicin (**12**) could be detected (Supplementary Figure S22). These results also align with *in vivo* findings, where engineered G001 strains, such as MAG304, accumulate *N,N*-dimethyl-13-deoxydaunorubicin **9**) and *N,N*-dimethyl-13-dihydrodaunorubicin (**10**) with trace amounts of *N,N*-dimethyldaunorubicin (**11**). The results suggest that the activity of DoxA with *N,N*-dimethylated substrates likely represents a limiting factor in the biosynthesis of *N,N*-dimethylated anthracyclines (see Discussion).

### 3.5 *N,N*-dimethyldoxorubicin is toxic to the producer strain

**FIGURE 6:**
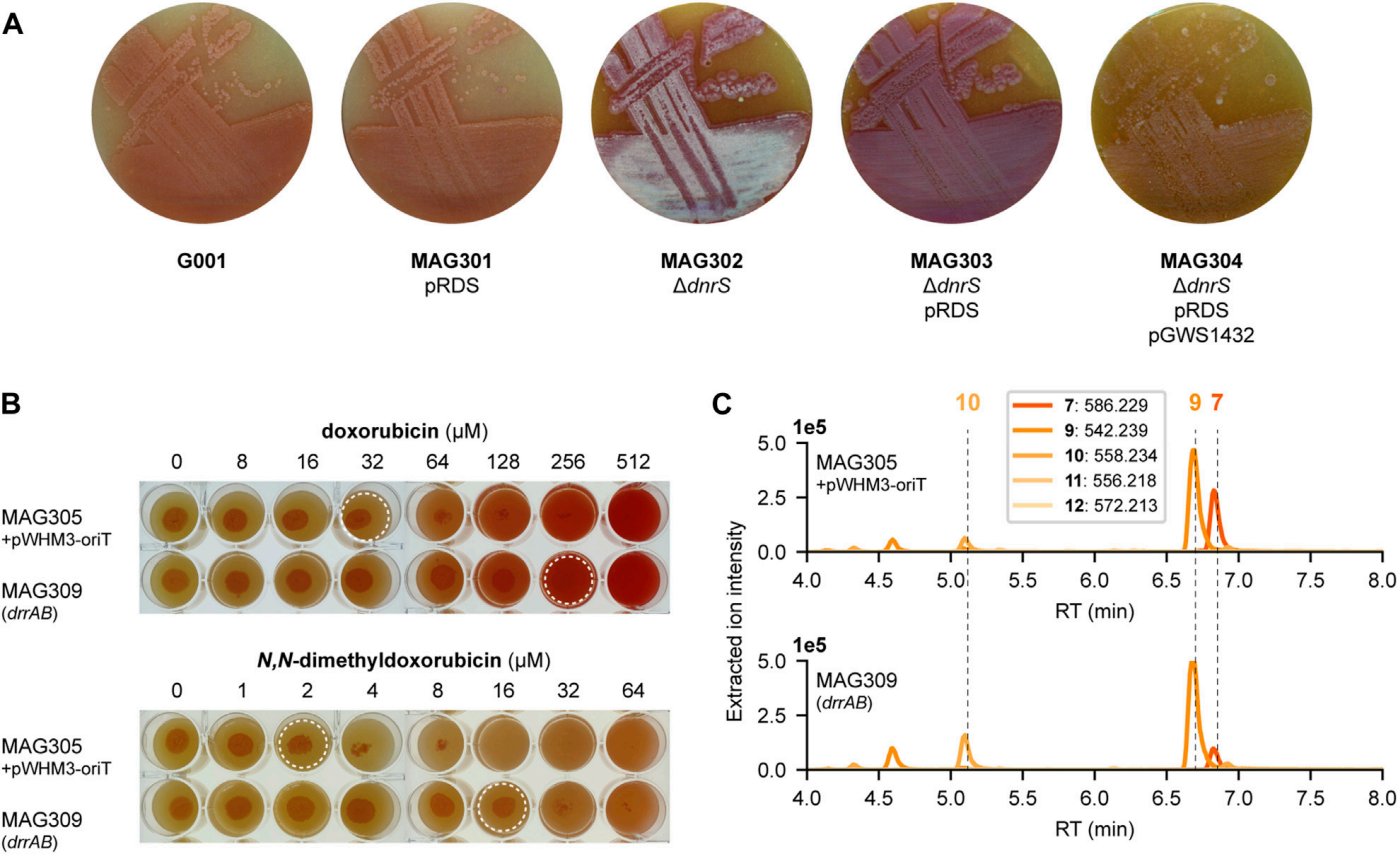
Toxicity of *N*,*N*-dimethyldoxorubicin. **(A)** G001, MAG301, MAG302, MAG303 and MAG304 were streaked on SFM agar plates (supplemented with 20 μg mL^−1^ thiostrepton for strains harbouring pRDS). The development is blocked by the production of anthracyclines. MAG304 exhibited the most pronounced inhibition of development. In contrast, deletion of the glycosyltransferase gene *dnrS* in MAG302 stimulated development. **(B)** MAG305 pWHM3-oriT and MAG309 (*drrAB*) were spotted on SFM agar plates supplemented with increasing concentrations of doxorubicin (**6**) or *N,N*-dimethyldoxorubicin (**12**). For each strain, 5 µL of spore or mycelium stock was spotted at a concentration of 1.0.10^4^ CFU per spot and the plates were incubated at 30 °C for 4 days. Dashed circles indicate the highest concentration that does not inhibit growth. For MAG305, the inhibitory concentration of *N,N*-dimethyldoxorubicin (**12**) is 16-fold lower than that of doxorubicin (**6**). Overexpression of *drrAB* increased resistance to both compounds eight-fold. **(C)** LC-MS analysis of crude extracts of MAG305 pWHM3-oriT and MAG309 (*drrAB*) cultivated in E1 medium. Extracted ion chromatograms showing the mass peaks [M + H]^+^ of compounds **7**–**12**. Overexpression of *drrAB* resulted in a 3.7-fold increased production of *N,N*-dimethyldaunorubicin (**11**).

During the construction of the engineered G001 strains, we noticed that the development of the strains was blocked, most likely caused by the production of cytotoxic anthracyclines. On SFM agar plates, G001 and MAG301 (G001 pRDS) exhibited a distinctive ‘bald’ phenotype, characterised by the absence of aerial hyphae and spores (Figure 6A). Notably, the deletion of the glycosyltransferase gene *dnrS* (MAG302) led to in the production of white aerial hyphae. However, upon introduction of the aclarubicin glycosyltransferase and methyltransferase genes (MAG303) the strain reverted to the ‘bald’ phenotype without aerial hyphae production. The introduction of the methylesterase gene *rdmC* (MAG304) further crippled the strain, evident by the reduction in colony size. We hypothesised that product toxicity could inhibit the development of the strains and consequently impact productivity. Therefore, we conducted a microbial inhibition assay involving doxorubicin, *N,N*-dimethyldoxorubicin (**12**) and two precursors.

To investigate the resistance of G001, 5 µL of mycelium stock was spotted at a concentration of 1.0.10^4^ CFU per spot on SFM agar plates with increasing concentration of 13-deoxydaunorubicin **3**), doxorubicin **6**), *N,N*-dimethyl-13-deoxydaunorubicin **9**) or *N,N*-dimethyldoxorubicin (**12**). After 3 days of incubation at 30 °C, growth was examined visually. Surprisingly, G001 exhibited no growth at 5 μg mL^−1^ *N,N*-dimethyldoxorubicin (**12**), whereas its growth was uninhibited at 25 μg mL^−1^ doxorubicin (Supplementary Figure S5). Furthermore, the main product of the engineered strain MAG304, *N,N*-dimethyl-13-deoxydaunorubicin **9**), inhibited the growth of G001 at a concentration of 10 μg mL^−1^, whereas no growth inhibition was observed when exposed to 25 μg mL^−1^ 13-deoxydaunorubicin **3**). These results suggest that the toxicity of the produced compounds may inhibit the biosynthesis of *N,N*-dimethylated anthracyclines.

#### 3.5.1 Enhanced resistance to *N*,*N*-dimethyldoxorubicin through overexpression of doxorubicin transporter genes

The doxorubicin BGC harbours several resistance genes, including the ABC-transporter genes *drrA* and *drrB*. Overexpression of these transporter genes may alleviate the toxicity associated with *N,N*-dimethylated anthracyclines. The coding region of *drrA*-*drrB* was positioned downstream of the constitutive ermE* promoter (Bibb et al., 1985), and cloned into the multicopy vector pWHM3-oriT to generate pGWS1437 (Supplementary Figure S1). pWHM3-oriT is a derivative of pWHM3 (Vara et al., 1989) that harbours *oriT* to allow for its conjugative transfer. Subsequently, pGWS1437 was introduced to MAG305 (G001 Δ*dnrS* pGWS1433), via conjugation, resulting in strain MAG309.

To investigate the resistance of MAG305 pWHM3-oriT and MAG309 (*drrAB*), 5 µL of mycelium stock was spotted at a concentration of 1.0.10^4^ CFU per spot on SFM agar plates with increasing concentration of doxorubicin **6**) or *N,N*-dimethyldoxorubicin (**12**). After 4 days of incubation at 30°C, growth was examined visually. For MAG305, the inhibitory concentration of *N,N*-dimethyldoxorubicin was 16-fold lower than that of doxorubicin. Overexpression of the *drrAB* genes in MAG309 resulted in an eight-fold increase in resistance to both doxorubicin (32–256 µM) and *N,N*-dimethyldoxorubicin (2–16 µM) (Figure 6B). Although the resistance of MAG309 to *N,N*-dimethyldoxorubicin (16 µM) remained two-fold lower than that of the control strain to doxorubicin (32 µM), this enhanced resistance may increase *N,N*-dimethylated anthracycline production. MAG305 pWHM3-oriT and MAG309 (*drrAB*) were cultivated in E1 medium, and the metabolite profile was analysed and annotated in a similar manner as described above. In both strains, the main peak in the chromatogram corresponded to *N,N*-dimethyl-13-deoxydaunorubicin **9**), and *N,N*-dimethyldoxorubicin (**12**) could not be detected (Figure 6C). However, overexpression of *drrAB* resulted in a relative increase of 2.1-fold, 4.9-fold and 3.7-fold in the production of *N*,*N*-dimethyl-13-deoxydaunorubicin **9**), *N,N*-dimethyl-13-dihydrodaunorubicin (**10**) and *N*,*N*-dimethyldaunorubicin (**11**), respectively (*n* = 3). Additionally, the peak area of ɛ-rhodomycin T **7**) was 1.3-fold decreased. These results indicate that the overexpression of *drrAB* pushed the reaction more toward *N,N*-dimethyldaunorubicin (**11**). However, it is important to note that the tailoring reactions catalysed by DoxA require optimisation for efficient biosynthesis of *N,N*-dimethyldaunorubicin (**11**) and *N,N*-dimethyldoxorubicin (**12**).

## 4 Discussion

The aim of this study was the biosynthesis of two anthracyclines *N*,*N*-dimethyldaunorubicin (**11**) and *N*,*N*-dimethyldoxorubicin (**12**), because of their significant therapeutic potential (Qiao et al., 2020; van Gelder et al., 2023). To achieve this goal, a combinatorial engineering approach was adopted, involving the introduction of genes from the aclarubicin and rhodomycin BGCs into the industrial doxorubicin overproducer G001. This strategy successfully led to the biosynthesis of *N*,*N*-dimethyldaunorubicin (**11**). However, it resulted in low yields of *N,N*-dimethyldaunorubicin (**11**) with no detection of its downstream derivative *N,N*-dimethyldoxorubicin (**12**). Subsequent attempts to optimise the final tailoring reactions catalysed by the cytochrome P450 monooxygenase DoxA proved challenging.

Firstly, we introduced the genes encoding the sugar *N*-methyltransferases AclP and AknX2 and the glycosyltransferases AknS and AknT from the aclarubicin biosynthetic pathway into G001 using the multicopy plasmid pRDS (Han et al., 2011). Introduction of pRDS into G001 led to the successful incorporation of l-rhodosamine onto ɛ-rhodomycinone (**16**) (Figure 3). Subsequent deletion of the native glycosyltransferase gene *dnrS* abolished production of daunorubicin **5**), a by-product in this context (Figure 3). Analysis of the produced metabolites revealed that the 15-methylesterase activity, catalysed by DnrP in the native doxorubicin pathway, did not occur in the engineered strain (Figure 3). Instead, ɛ-rhodomycin T **7**) was directly 4-*O*-methylated by the moonlighting activity of DnrK, yielding 4-methoxy-ɛ-rhodomycin T (**13**) (Grocholski et al., 2015). Furthermore, incomplete 11-hydroxylation activity by DnrF resulted in the by-products aclacinomycin T (**14**) and 4-*O*-methyl-aclacinomytin T (**15**).

Subsequently, we introduced the gene encoding the DnrP homolog RdmC from the rhodomycin biosynthetic pathway into the engineered strain. RdmC catalyses the conversion of ɛ-rhodomycin T **7**) into 15-demethyl-ɛ-rhodomycin T **8**) in the rhodomycin pathway (Grocholski et al., 2015). Introduction of *rdmC* to the engineered strain resulted in the desired 15-demethylation activity, and subsequent 4-*O*-methylation and 10-carboxylation by DnrK (Figure 4). Notably, this resulted in the production of the targeted compounds, *N,N*-dimethyldaunorubicin (**11**). While the engineered strain produced *N,N*-dimethyldaunorubicin (**11**), it was in limited quantities, and the final step toward *N,N*-dimethyldoxorubicin (**12**) was not achieved.

In the native doxorubicin pathway, the multistep conversion of 13-deoxydaunorubicin **3**) to doxorubicin **6**) is catalysed by DoxA. Notably, the conversion from daunorubicin **5**) to doxorubicin **6**) is more than 100-fold less efficient compared to the previous two steps (Walczak et al., 1999). Given the inherent inefficiency of the 14-hydroxylation, even with the natural substrate, it is not surprising that the corresponding 14-hydroxylation of the unnatural substrate *N,N*-dimethyldaunorubicin (**11**) is challenging. However, the accumulation of *N,N*-dimethyl-13-deoxydaunorubicin **9**) and *N,N*-dimethyl-13-dihydrodaunorubicin (**10**) in the engineered strain is unexpected considering that the conversion to daunorubicin **5**) is complete in the parental strain. Consequently, DoxA constitutes a potential bottleneck in the proposed biosynthetic pathway.

We confirmed that DoxA was expressed in significant quantities in the engineered strain. Quantitative proteomics demonstrated the abundance of DoxA in the engineered strain MAG304, suggesting that the protein level of DoxA is not a limiting factor (Figure 5A). In contrast to the previous engineering steps, no heterologous DoxA enzyme is known to accept *N,N*-dimethylated substrates. In fact, the DoxA enzyme is unique to the doxorubicin biosynthetic pathway. Nevertheless, we decided to express the genes encoding close DoxA homologs from *S. bellus* and *S. coeruleorubidus* in the engineered strain. Unsurprisingly, this effort did not result in improved productivity (Figure 5B).

To confirm that the activity of DoxA is a key bottleneck in the production of *N,N*-dimethylated anthracyclines, we conducted enzymatic assays of DoxA with both natural and *N,N*-dimethylated substrates. The reaction products indicated that while DoxA efficiently converts the natural substrate 13-deoxydaunorubicin **3**) to daunorubicin **5**), the conversion of *N,N*-dimethyl-13-deoxydaunorubicin **9**) is very inefficient (Figures 5C,D). Moreover, DoxA could not convert *N,N*-dimethyldaunorubicin (**11**) to *N,N*-dimethyldoxorubicin (**12**) (Supplementary Figure S22). Taken together, these findings suggest that DoxA is inhibited by the *N,N*-dimethyl moiety of the unnatural substrates. Further studies should be conducted to confirm this hypothesis. Rational engineering of DoxA may be required to optimize the activity to identify mutant DoxA variants with enhanced enzymatic activity for conversion of *N,N*-dimethylated substrates.

This study also highlighted the inherent challenge of cytotoxicity associated with anthracycline production in the producer strain. *N,N*-dimethylated anthracyclines proved to assert a stronger cytotoxic effect than the natural variants (Supplementary Figure S5). Overexpression of *drrAB* in the engineered strain resulted in an eight-fold increase in resistance to both doxorubicin **6**) and *N,N*-dimethyldoxorubicin (**12**) (Figure 6). This improved resistance pushed the pathway more toward *N,N*-dimethyldaunorubicin (**11**), although the productivity was still low (Figure 6).

The efficient production of *N,N*-dimethyl-13-deoxydaunorubicin **9**) by MAG304 provides a promising outlook for biosynthesis of *N,N*-dimethylated anthracyclines. Further strain development in terms of DoxA activity and improved toxicity could provide sufficient productivity. In industry, doxorubicin is mainly produced semi-synthetically from daunorubicin (Lown, 1993). Similarly, it would be possible to produce *N,N*-dimethyldoxorubicin semi-synthetically if productivity of *N,N*-dimethyldaunorubicin is achieved.

In conclusion, *N,N*-dimethylated anthracyclines represent promising alternatives for conventional anticancer drugs, offering reduced cardiotoxic risks (Qiao et al., 2020; van Gelder et al., 2023). This study demonstrates the potential for biosynthesis of *N,N*-dimethylated anthracyclines via combinatorial biosynthesis. While we successfully produced *N,N*-dimethyldaunorubicin (**11**), future work should focus on optimizing the cytochrome P450 monooxygenase DoxA, responsible for the final three oxidation reactions in the engineered pathway.

## Data Availability

Metabolomics data is available via MassIVE (accession MSV000093884). Proteomics data is available via ProteomeXchange (accession PXD048604).
