# Supplementary material for: Metabolic engineering of Streptomyces peucetius for biosynthesis of N,N-dimethylated anthracyclines
Source: Front Bioeng Biotechnol. 2024 Feb 28;12:1363803. doi: 10.3389/fbioe.2024.1363803 (PMC10936713; doi:10.3389/fbioe.2024.1363803)
Supplement: Supplementary file 1 [file DataSheet1.PDF]

Supplementary Material

Metabolic engineering of *Streptomyces peucetius* for biosynthesis of *N*,*N*-dimethylated anthracyclines

Mandy B. Hulst[1], Le Zhang[1], Helga U. van der Heul[1], Chao Du[1], Somayah S.M.A. Elsayed[1], Arina Koroleva[2], Thadee Grocholski[2], Dennis P. A. Wander[3], Mikko Metsä-Ketelä[2], Jacques J. C. Neefjes[3] and Gilles P. van Wezel[1*]

[1]Institute of Biology, Leiden University, Sylviusweg 72, 2333 BE Leiden, the Netherlands

[2]Department of Life Technologies, University of Turku, FIN-20014 Turku, Finland

[3]Department of Cell and Chemical Biology, ONCODE Institute, Leiden University Medical Centre LUMC, Einthovenweg 20, 2333 ZC Leiden, The Netherlands

*** Correspondence:**
Gilles P. van Wezel
g.wezel@biology.leidenuniv.nl

1 Supplementary Tables

Table S1. Plasmids used in this study.

Plasmid	Description	References
pWHM3	*E. coli*/*Streptomyces* shuttle vector, high copy number in *E. coli*, Amp^R, Thio^R	Vara et al., 1989
pWHM3-oriT	pWHM3-derivative harbouring *oriT* site for conjugative transfer in the NdeI site, Amp^R, Thio^R	Wu et al., 2019
pSET152	*E. coli*/*Streptomyces* shuttle vector, harbouring *attP* site and integrase for phage φC31 for stable integration into the chromosomal *attB* site of *Streptomyces*, Apra^R	Bierman et al., 1992
pMS82	*E. coli*/*Streptomyces* shuttle vector, harbouring *attP* site and integrase for phage φBT1 for stable integration into the chromosomal *attB* site of *Streptomyces*, Hyg^R	Gregory et al., 2003
pUWLCRE	Cre recombinase expression construct, Amp^R, Thio^R	Fedoryshyn et al., 2008
pRDS	pWHM3-derivative with aclarubicin, doxorubicin and rhodomycin biosynthetic genes, Amp^R, Thio^R	Han et al., 2011
pBAD/HisB	*E. coli* construct for protein expression with His-tag, Amp^R	Invitrogen
pBAD/HisB-rdmC	pBAD/HisB-derivative with *rdmC* from *S. purpurascens* ATCC 25489, Amp^R	Grocholski et al., 2015
pBAD/HisB-doxA	pBAD/HisB-derivative with *doxA* from *S. peucetius* ATCC 27952, Amp^R	Koroleva et al., 2024
pBAD/HisB-dnrV	pBAD/HisB-derivative with *dnrV* from *S. peucetius* ATCC 27952, Amp^R	Koroleva et al., 2024
pBAD/HisB-fdx4	pBAD/HisB-derivative with *fdx4* from *S. peucetius* ATCC 27952, Amp^R	Koroleva et al., 2024
pBAD/HisB-sfr	pBAD/HisB-derivative with spinach ferredoxin reductase gene *sfr*, Amp^R	Koroleva et al., 2024
pUCK_L3S1P47	pUCK-derivative with the synthetic L3S1P47 terminator, Kan^R	Chen et al., 2013
pGWS1431	pWHM3-derivative with the flanking regions of *S. peucetius dnrS* interspersed with the Apra^R-*loxP* cassette, Amp^R, Thio^R, Apra^R	This work
pGWS1432	pSET152-derivative with the coding region of *S. purpurascens rdmC* under control of the *ermE** promoter, Apra^R	This work
pGWS1433	pSET152-derivative with the expression cassette of pRDS and coding region of *rdmC* under control of the R15 RBS and L3S1P47 terminator, Apra^R	This work
pGWS1434	pMS82-derivative with codon optimized *S. peucetius doxA* under control of the P7 promoter, R9 RBS and *aph* terminator, Hyg^R	This work
pGWS1435	pMS82-derivative with codon optimized *S. bellus doxA* under control of the P7 promoter, R9 RBS and *aph* terminator, Hyg^R	This work

pGWS1436	pMS82-derivative with codon optimized *S. coeruleorubidus doxA* under control of the P7 promoter, R9 RBS and *aph* terminator, Hyg^R	This work
pGWS1437	pWHM3-oriT-derivative with the coding region of *S. peucetius drrAB* under control of the *ermE** promoter, Amp^R, $Thio^R$	This work

Table S2. Primers used in this study.

Primer	Sequence (5' to 3')*
MH301	GATC**AAGCTT**CCCGCAGGAGCACGTTCTCG
MH302	GATCGAAGTTATCGCGCATC**TCTAGA**ACCCTCCCTGGGTCACTTCTG
MH303	GATCGAAGTTATCCATCACC**TCTAGA**TGGAGTACGGCCAGGTAGAG
MH304	GATC**GAATTC**ATCGTCATCGAGCACAATC
MH305	CCCGGTTTCACGCACATGGC
MH306	GTCCCGGCAGATCAGCGTGG
MH307	GATCGAATTC**CATATG**ATGTCCGAACGCATCGTGCCGAG
MH308	GATC**TCTAGA**CAGCCAAGCTTCATCGTCTC
MH309	ATGTTCTTTCCTGCGTTATCTCTAAGTAAGGAGTGTCCATATGTCCGAACGCATCGTGCCGAGCG
MH310	GGGAGGCCTTTTTTCGAAAATCAGGCCGCCGAGCGGGTGT
MH311	ACACCCGCTCGGCGGCCTGATTTTCGAAAAAAGGCCTCCCAAATC
MH312	ATGGACACTCCTTACTTAGAGATAACGCAGGAAAGAACATGTGAGCAAAAGGCC
MH313	AGCCGGCCGCGCGGGACTGATCTAAGTAAGGAGTGTCCATATG
MH314	ACGGCCAGTGCCAAGCTTGGGCTGCAGGTCGACTCTAGAGGGATCCTTTTGTTGCTATAAAAAAAGGCCCC
MH315	ATAACAATTTCACACAGGAAACAGCTATGACATGATTACGGAATTCCTGGAGGACGGACC
MH316	ATGGACACTCCTTACTTAGATCAGTCCCGCGCGGCCGGCTGCG
MH317	GGCGGGCGACGCGGGACCGG
MH318	GAATTCAACGGAACAGCCGGCGTCGGTGAACGCGG
MH319	CCGGCTGTTCCGTTGAATTCCTGGAGGACGGACCCACCGGCCGCG
MH320	GTGCCAAGCTTGGGCTGCAGGTCGACTCTAGAGGGATCC
MH321	GATCGAATTC**CATATG**AACACGCAGCCGACACG
MH322	GATCAAGCTT**TCTAGA**CTCACACCCCCTCAACGACG
M13_R	AGCGGATAACAATTTCACACAGG

* Restriction sites used for cloning are presented in boldface: GAATTC, EcoRI; CATATG, NdeI; AAGCTT, HindIII; TCTAGA, XbaI; GGATCC, BamHI; GGTACC, KpnI.

Table S3. LC-MS data used to identify compounds **1**–**16** in the extracts of the tested strains.

#	Name	Chemical formula	Calculated *m/z*, adduct ion	Observed *m/z*	RT (min)	Source
1	rhodomycin D	$C_{28}H_{31}NO_{11}$	558.1975, $[M+H]^+$	N.D.	N.D.	N.D.
2	15-demethoxy-rhodomycin D	$C_{27}H_{29}NO_{11}$	544.1819, $[M+H]^+$	N.D.	N.D.	N.D.
3	13-deoxy-daunorubicin	$C_{27}H_{31}NO_9$	514.2077, $[M+H]^+$; 367.1182, $[M+H\text{-sugar-}H_2O]^+$	514.2071; 367.1174	6.4	reference compound (van Gelder et al., 2023)
4	13-dihydro-daunorubicin	$C_{27}H_{31}NO_{10}$	530.2026, $[M+H]^+$; 383.1131, $[M+H\text{-sugar}]^+$	530.2017; 383.1126	4.9	crude extract G001
5	daunorubicin	$C_{27}H_{29}NO_{10}$	528.1870, $[M+H]^+$; 363.0868, $[M+H\text{-sugar-}H_2O]^+$	528.1862; 363.0864	5.5	Sanofi BV
6	doxorubicin	$C_{27}H_{29}NO_{11}$	544.1819, $[M+H]^+$; 379.0818, $[M+H\text{-sugar-}H_2O]^+$	544.1816; 379.0812	4.6	Accord Healthcare Limited
7	ε-rhodomycin T	$C_{30}H_{35}NO_{11}$	586.2288, $[M+H]^+$; 393.0974, $[M+H\text{-sugar-}H_2O]^+$	586.2285; 393.0970	6.9	crude extract MAG301
8	15-demethoxy-ε-rhodomycin T	$C_{29}H_{33}NO_{11}$	572.2132, $[M+H]^+$	N.D.	N.D.	N.D.
9	*N*,*N*-dimethyl-13-deoxydaunorubicin	$C_{29}H_{35}NO_9$	542.2390, $[M+H]^+$; 349.1076, $[M+H\text{-sugar-}H_2O]^+$	542.2385; 349.1071	6.7	reference compound (van Gelder et al., 2023)
10	*N*,*N*-dimethyl-13-dihydrodaunorubicin	$C_{29}H_{35}NO_{10}$	558.2339, $[M+H]^+$; 383.1131, $[M+H\text{-sugar}]^+$	558.2333; 383.1124	5.1	crude extract MAG304
11	*N*,*N*-dimethyl-daunorubicin	$C_{29}H_{33}NO_{10}$	556.2183, $[M+H]^+$; 363.0868, $[M+H\text{-sugar-}H_2O]^+$	556.2177; 363.0861	5.7	reference compound (van Gelder et al., 2023)
12	*N*,*N*-dimethyl-doxorubicin	$C_{29}H_{33}NO_{11}$	572.2132, $[M+H]^+$; 379.0818, $[M+H\text{-sugar-}H_2O]^+$	N.D.	4.7	reference compound (Qiao et al., 2020)
13	4-methoxy-ε-rhodomycin T	$C_{31}H_{37}NO_{11}$	600.2445, $[M+H]^+$; 407.1131, $[M+H\text{-sugar-}H_2O]^+$	600.2441; 407.1127	6.4	crude extract MAG301
14	aclacinomycin T	$C_{30}H_{35}NO_{10}$	570.2339, $[M+H]^+$; 377.1025, $[M+H\text{-sugar-}H_2O]^+$	570.2334; 377.1021	6.4	crude extract MAG301
15	4-methoxy-aclacinomycin T	$C_{31}H_{37}NO_{10}$	584.2496, $[M+H]^+$; 391.1182, $[M+H\text{-sugar-}H_2O]^+$	584.2492; 391.1174	6.0	crude extract MAG301
16	ε-rhodomycinone	$C_{22}H_{20}O_9$	451.1005, $[M+Na]^+$; 361.0712, $[M+H\text{-}2H_2O\text{-}2CH_3OH]^+$	451.1006; 361.0707	9.5	crude extract G001

2 Supplementary Figures

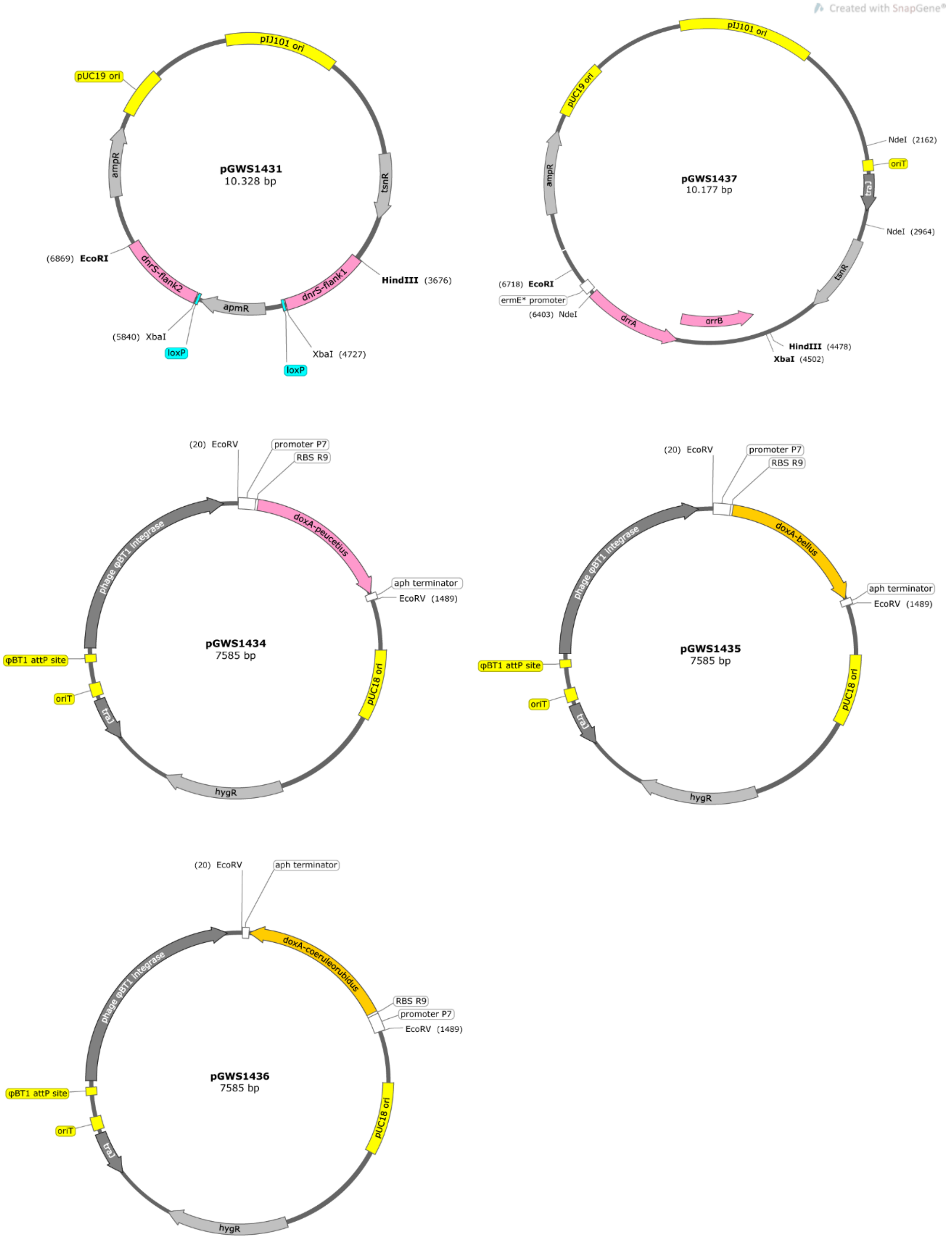

Figure S1. Plasmid maps of constructs used in this study.

Restriction sites used for cloning are indicated. Resistance cassettes are abbreviated as: *ampR*, ampicillin; *apmR*, apramycin; *hygR*, hygromycin; *tsnR*, thiostrepton. Biosynthetic genes are coloured by source organism: *S. peucetius*, pink; *S. galilaeus*, blue; *S. venezuelae*, purple; *S. purpurascens*, green; other, orange. Plasmid maps were generated using SnapGene 6.0.

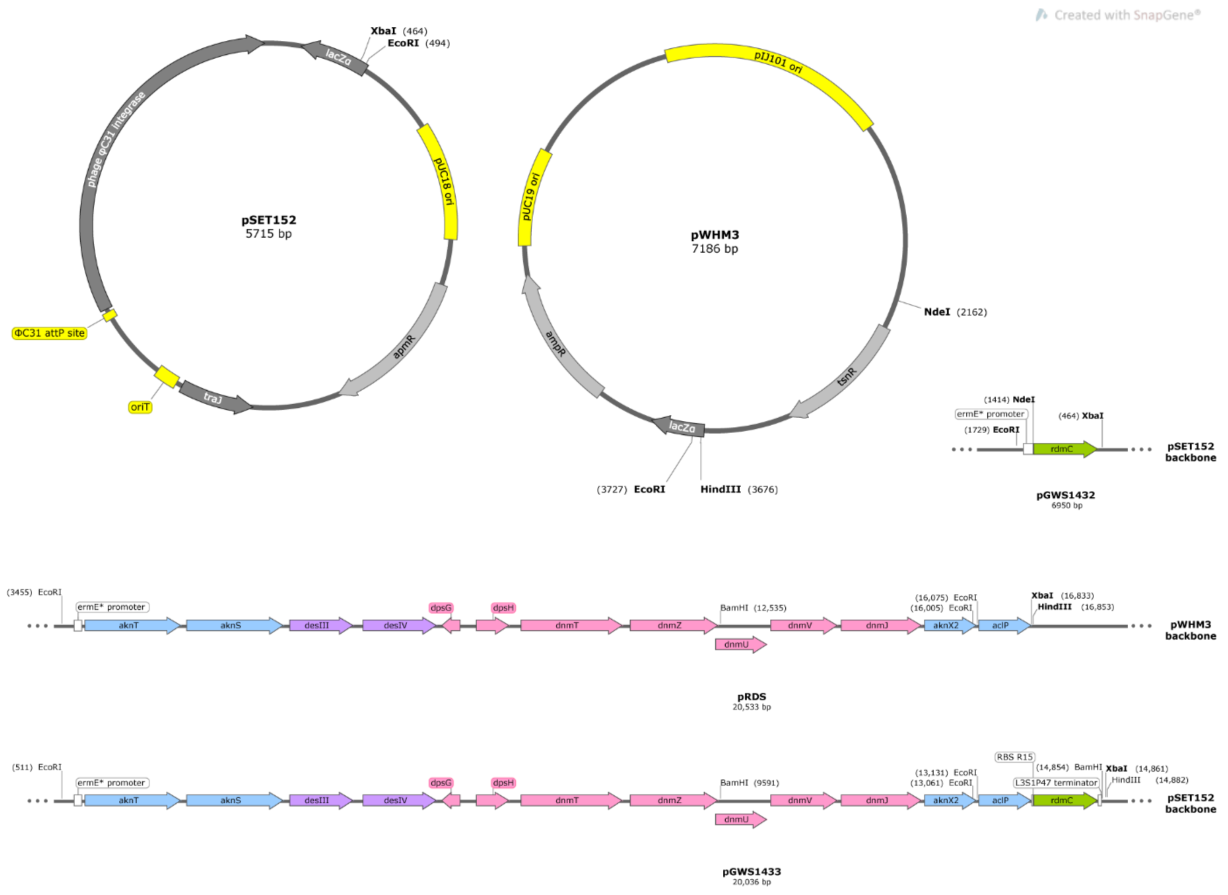

Figure S1. Continued

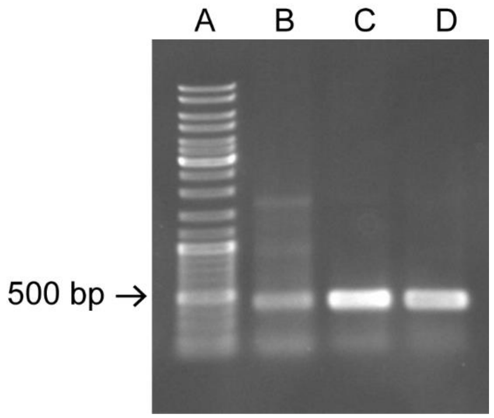

Figure S2. PCR analysis confirms the successful deletion of *dnrS* in G001.

Gel electrophoresis of PCR products of primers MH305 and MH306 on genomic DNA of three clones of G001 Δ*dnrS*. The amplification of the target DNA fragment of 489 bp confirms the successful knock out of *dnrS* in all three clones. G001 *ΔdnrS* clone 2 was selected. Lane A: GeneRuler DNA Ladder Mix. Lane B–D: G001 Δ*dnrS* clone 1–3, respectively.

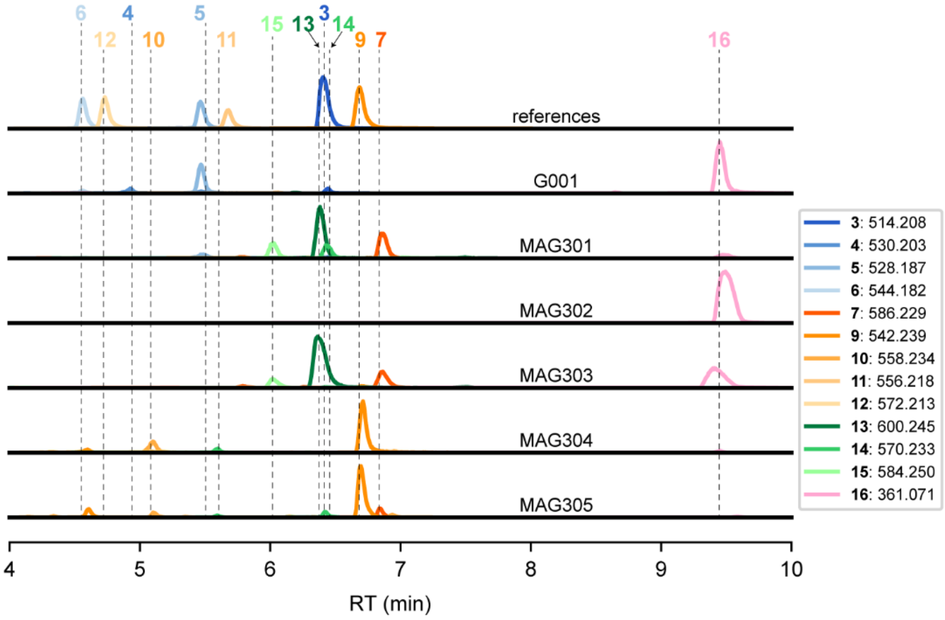

Figure S3. Analysis of engineered strains by LC-MS/MS.

LC-MS analysis of crude extracts of G001 and engineered strains MAG301 (G001 pRDS), MAG302 (G001 Δ*dnrS*), MAG303 (G001 Δ*dnrS* pRDS), MAG304 (G001 Δ*dnrS* pRDS pGWS1432) and MAG305 (G001 Δ*dnrS* pGWS1433) cultivated in E1 medium with added HP20 resin. Extracted ion chromatograms showing the mass peaks $[M+H]^+$ of compounds **3**–**7** and **9**–**15**, and the mass peaks $[M+H\text{-}2H_2O\text{-}2CH_3OH]^+$ of compound **16**.

```
Q59971.1                MSGEAPRVAVDPFSCPMMTMQRKPEVHDAFREAGPVVEVNAPAGGPAWVITDDALAREVL       60
WP_167537468.1          -------------------MQRKPEVHDAFREAGPVVEVNAPAGGPAWVITDDALAREVL       41
ATW50545.1              MSGEAPRVAVDPFACPMMTMQRKPEVHDAFREAGPVVEVNAPAGGPAWVITDDALAREVL       60
Q9ZAU3.1                -------MAVDPFACPMMTMQRKPEVHDAFREAGPVVEVNAPAGGPAWVITDDALAREVL       53
WP_274819524.1          -------------------MQRKPEVHDAFREAGPVVEVNAPAGGPAWVITDDALAREVL       41
WP_193507397.1          -------------------MQRKPEVHDAFREAGPVVEVNAPAGGPAWVITDDALAREVL       41
                                           *****************************************

Q59971.1                ADPRFVKDPDLAPTAWRGVDDGLDIPVPELRPFTLIAVDGEDHRRLRRIHAPAFNPRRLA       120
WP_167537468.1          ADPRFVKDPDLAPAAWRGVDDGLDIPVPELRPFTLIAVDGEAHRRLRRIHAPAFNPRRLA       101
ATW50545.1              ADPRFVKDPDLAPAAWRGVDDGLDIPVPELRPFTLIAVDGEAHRRLRRIHAPAFNPRRLA       120
Q9ZAU3.1                ADPRFVKDPDLAPAAWRGVDDGLDIPVPELRPFTLIAVDGEAHRRLRRIHAPAFNPRRLA       113
WP_274819524.1          ADPRFVKDPDLAPAAWRGVDDGLDIPVPELRPFTLIAVDGEAHRRLRRIHAPAFNPRRLA       101
WP_193507397.1          ADPRFVKDPDLAPAAWRGVDDGLDIPVPELRPFTLIAVDGEAHRRLRRIHAPAFNPRRLA       101
                        *************:*************************** ******************

Q59971.1                ERTDRIAAIADRLLTELADSSDRSGEPAELIGGFAYHFPLLVICELLGVPVTDPAMAREA       180
WP_167537468.1          ERTDRIAAIAGRLLTELADASGRSGKPAELIGGFAYHFPLLVICELLGVPVTDPAMAREA       161
ATW50545.1              ERTDRIAAIAGRLLTELADASGRSGKPAELIGGFAYHFPLLVICELLGVPVTDPAMAREA       180
Q9ZAU3.1                ERTDRIAAIAGRLLTELADASGRSGKPAELIGGFAYHFPLLVICELLGVPVTDPAMAREA       173
WP_274819524.1          ERTDRIAAIAGRLLTELADASGRSGKPAELIGGFAYHFPLLVICELLGVPVTDPAMAREA       161
WP_193507397.1          ERTDRIAAIAGRLLTELADTSGRSGKPAELIGGFAYHFPLLVICELLGVPVTDPAMAREA       161
                        **********.********:*.***:**********************************

Q59971.1                VGVLKALGLGGPQSAGGDGTDPAGDVPDTSALESLLLEAVHAARRKDTRTMTRVLYERAQ       240
WP_167537468.1          VSVLKALGLGGPQSGGGDGTDPAGDMPDTSALESLLLEAVHSARRNDTPTMTRVLYERAQ       221
ATW50545.1              VSVLKALGLGGPQSGGGDGTDPAGGVPDTSALESLLLEAVHSARRNDTPTMTRVLYERAQ       240
Q9ZAU3.1                VSVLKALGLGGPQSGGGDGTDPAGGVPDTSALESLLLEAVHSARRNDTPTMTRVLYERAQ       233
WP_274819524.1          VSVLKALGLGGPQSGGGDGTDPAGGVPDTSALESLLLEAVHSARRNDTRTMTRVLYERAQ       221
WP_193507397.1          VSVLKALGLGGPQSGGGDGTDPAGGVPDTSALESLLLEAVHSARRNDTPTMTRVLYERAQ       221
                        *.************.*********.:***************:***:** ***********

Q59971.1                AEFGSVSDDQLVYMITGLIFAGHDTTGSFLGFLLAEVLAGRLAADADGDAISRFVEEALR       300
WP_167537468.1          AEFGSVSDDQLVYMITGLIFAGHDTTGSFLGFLLAEVLAGRLAADADEDAVSRFVEEALR       281
ATW50545.1              AEFGSVSDDQLVYMITGLIFAGHDTTGSFLGFLLAEVLAGRLAADADEDAVSRFVEEALR       300
Q9ZAU3.1                AEFGSVSDDQLVYMITGLIFAGHDTTGSFLGFLLAEVLAGRLAADADEDAVSRFVEEALR       293
WP_274819524.1          AEFGSVSDDQLVYMITGLIFAGHDTTGSFLGFLLAEVLAGRLAADADEDAVSRFVEEALR       281
WP_193507397.1          AEFGSVSDDQLVYMITGLIFAGHDTTGSFLGFLLAEVLAGRLAADADEDAVSRFVEEALR       281
                        *********************************************** **:*********

Q59971.1                HHPPVPYTLWRFAATEVVIRGVRLPRGAPVLVDIEGTNTDGRHHDAPHAFHPDRPSRRRL       360
WP_167537468.1          YHPPVPYTLWRFAATEVTIGGVRLPRGAPVLVDIEGTNTDGRHHDAPHAFHPDRPSWRRL       341
ATW50545.1              YHPPVPYTLWRFAATEVTIGGVRLPRGAPVLVDIEGTNTDGRHHDAPHAFHPDRPSWRRL       360
Q9ZAU3.1                YHPPVPYTLWRFAATEVTIGGVRLPRGAPVLVDIEGTNTDGRHHDAPHAFHPDRPSWRRL       353
WP_274819524.1          YHPPVPYTLWRFAATEVTIGGVRLPRGAPVLVDIEGTNTDGRHHDAPHAFHPDRPSWRRL       341
WP_193507397.1          YHPPVPYTLWRFAATEVTIGGVRLPRGAPVLVDIEGTNTDGRHHDDPHAFHPDRPSWRRL       341
                        :****************.* ************************* ********** ***

Q59971.1                TFGDGPHYCIGEQLAQLESRTMIGVLRSRFPQARLAVPYEELRWCRKGAQTARLTDLPVW       420
WP_167537468.1          TFGDGPHYCIGEQLAQLESRTMIGVLRSRFPEARLAVPYDELRWSRKGAQTARLTELPVW       401
ATW50545.1              TFGDGPHYCIGEQLAQLESRTMIGVLRSRFPEARLAVPYDELRWCRKGAQTARLTELPVW       420
Q9ZAU3.1                TFGDGPHYCIGEQLAQLESRTMIGVLRSRFPEARLAVPYDELRWCRKGAQTARLTELPVW       413
WP_274819524.1          TFGDGPHYCIGEQLAQLESRTMIGVLRSRFPEARLAVPYDELRWSRKGAQTARLTELPVW       401
WP_193507397.1          TFGDGPHYCIGEQLAQLESRTMIGVLRSRFPEARLAVPYDELRWSRKGAQTARLTELPVW       401
                        *******************************:*******:****.**********:****

Q59971.1                LR           422   Streptomyces sp. C5
WP_167537468.1          LR           403   S. coeruleorubidus ATCC 13740
ATW50545.1              LR           422   S. peucetius var. caesius ATCC 27952
Q9ZAU3.1                LR           415   S. peucetius ATCC 29050
WP_274819524.1          LR           403   S. coeruleorubidus DM
WP_193507397.1          LR           403   S. bellus
                        **
```

Figure S4. Sequence alignment of DoxA with closest homologs found by an NCBI BLASTP search.

Protein sequences that share >95% identity with *S. peucetius* var. *caesius* ATCC 27952 DoxA (ATW50545.1) were aligned using Clustal Omega 1.2.4.

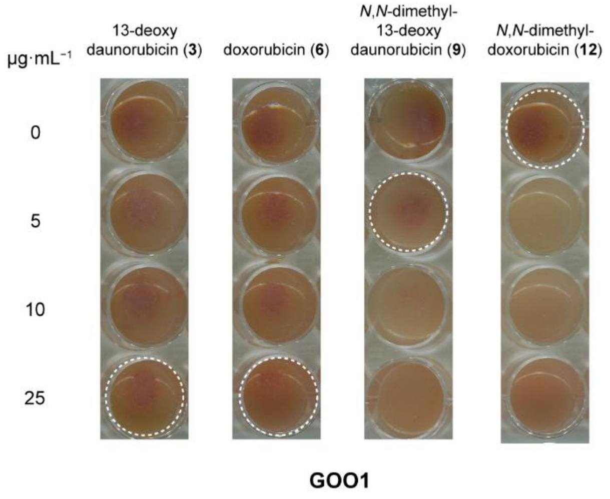

Figure S5. Toxicity of *N*,*N*-dimethyldoxorubicin.

G001 was spotted on SFM agar plates supplemented with increasing concentrations of 13-deoxydaunorubicin (**3**), doxorubicin (**6**), *N*,*N*-dimethyl-13-deoxydaunorubicin (**9**) and *N*,*N*-dimethyldoxorubicin (**12**) and incubated at 30 °C for 3 days. The highest concentration that supported growth is indicated by a white dashed circle.

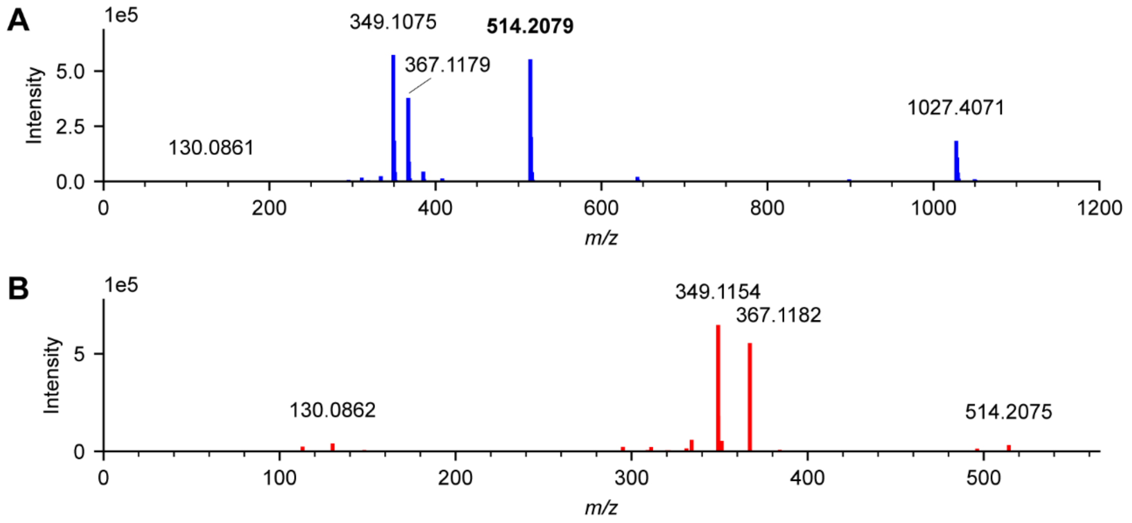

Figure S6. HRMS and MS/MS spectra of 13-deoxydaunorubicin (3) reference compound.

HRMS **(A)** and MS/MS **(B)** spectra were obtained from a reference compound of 13-deoxydaunorubicin (**3**).

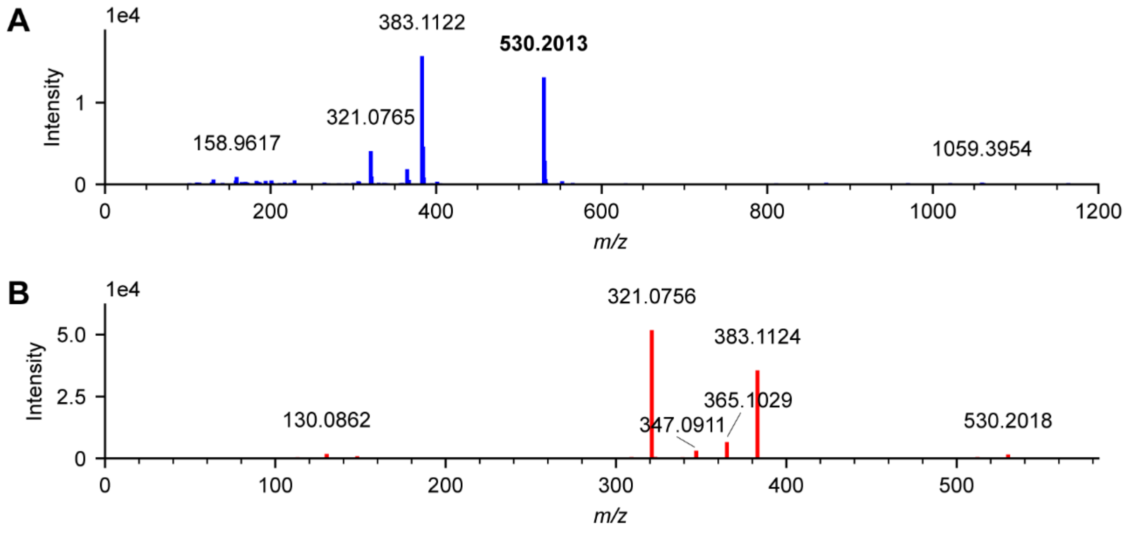

Figure S7. HRMS and MS/MS spectra of 13-dihydrodaunorubicin (4) annotated in crude extract.

HRMS **(A)** and MS/MS **(B)** spectra were obtained from a crude extract of G001.

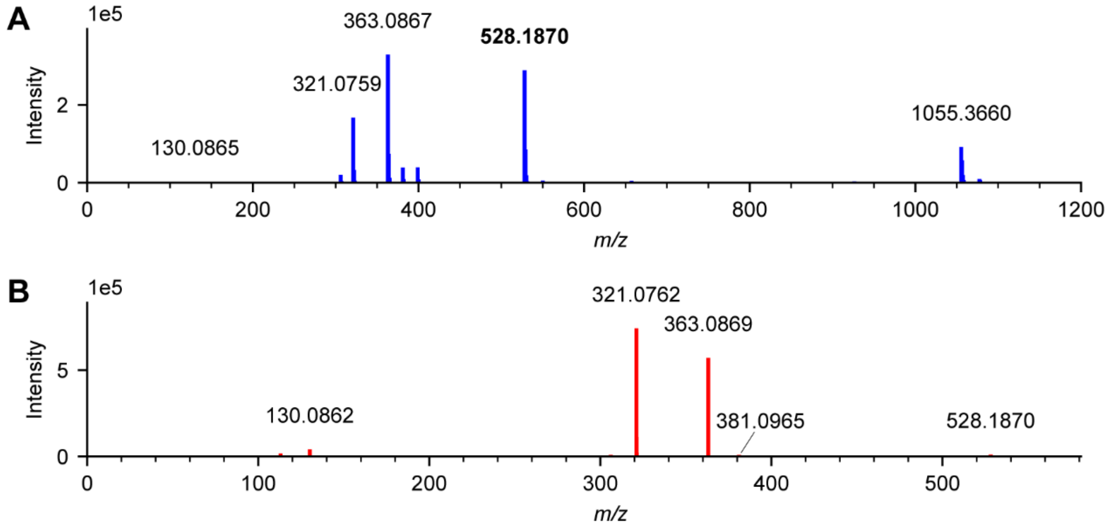

Figure S8. HRMS and MS/MS spectra of daunorubicin (5) reference compound.

HRMS **(A)** and MS/MS **(B)** spectra were obtained from a reference compound of daunorubicin (**5**).

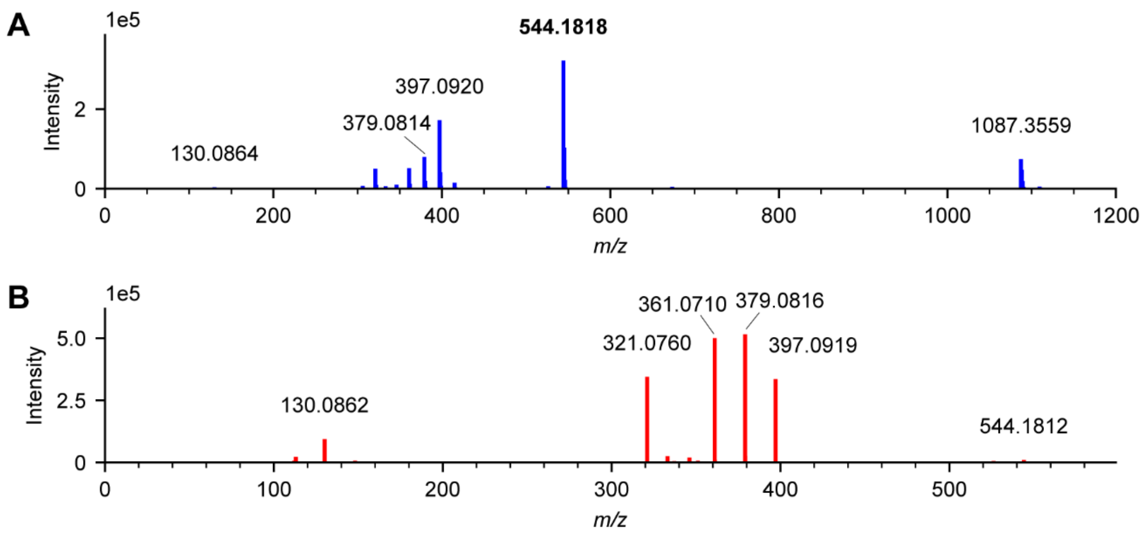

Figure S9. HRMS and MS/MS spectra of doxorubicin (6) reference compound.

HRMS **(A)** and MS/MS **(B)** spectra were obtained from a reference compound of doxorubicin (6).

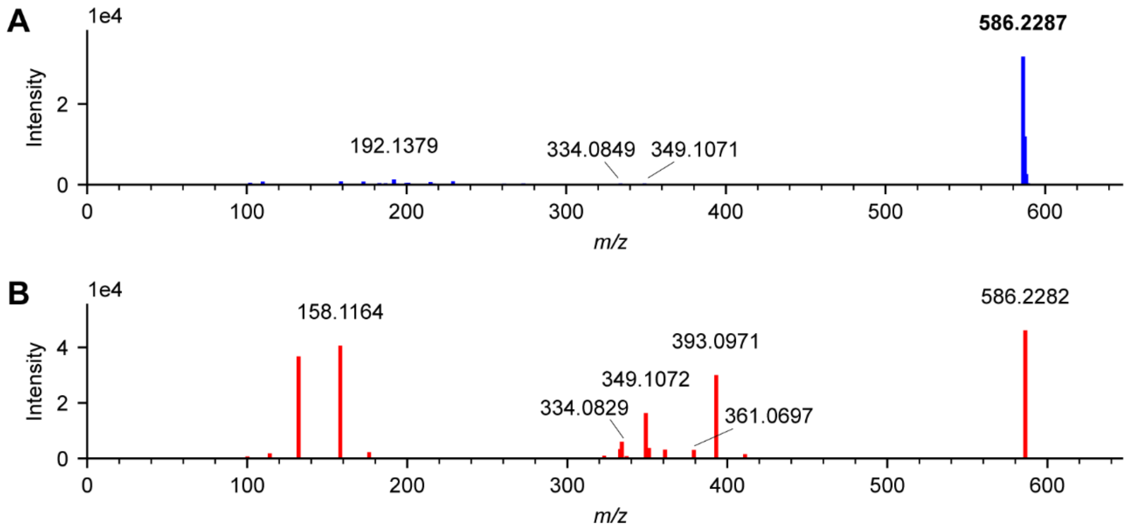

Figure S10. HRMS and MS/MS spectra of ε-rhodomycin T (7) from crude extract.

HRMS **(A)** and MS/MS **(B)** spectra were obtained from a crude extract of MAG301.

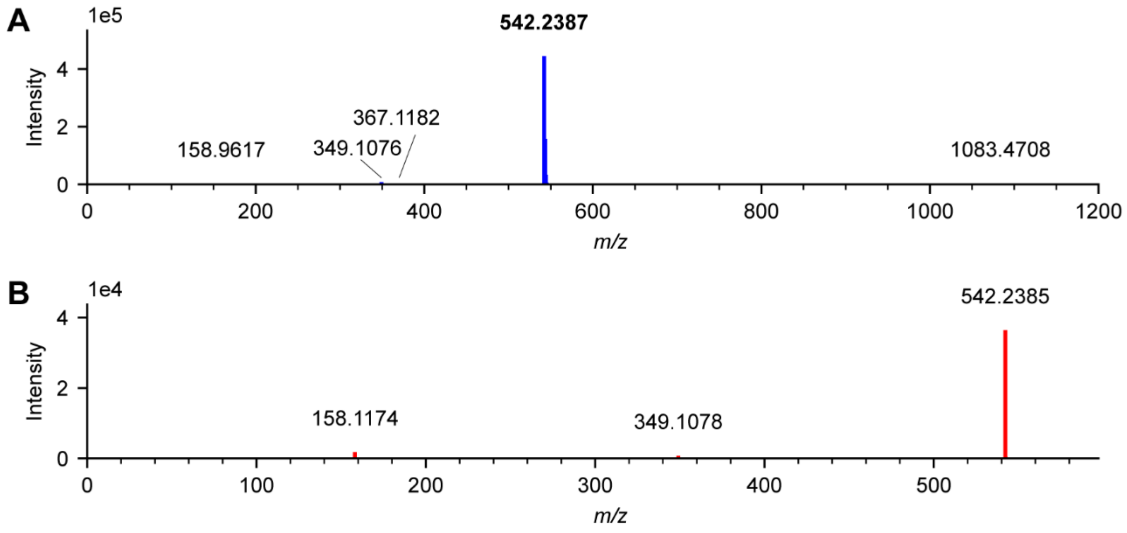

Figure S11. HRMS and MS/MS spectra of *N,N*-dimethyl-13-deoxydaunorubicin (9) reference compound.

HRMS **(A)** and MS/MS **(B)** spectra were obtained from a reference compound of *N*,*N*-dimethyl-13-deoxydaunorubicin (**9**).

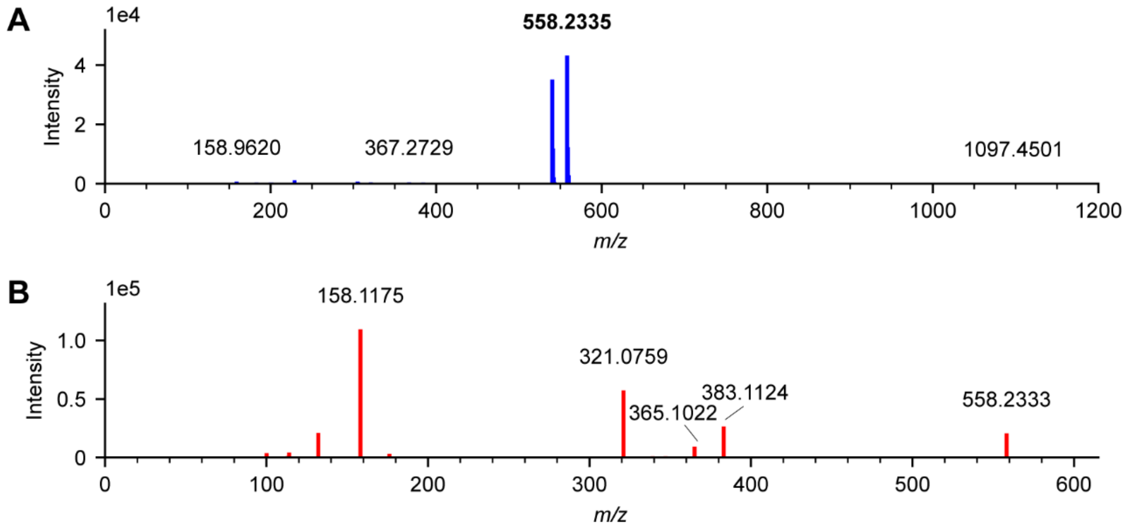

Figure S12. HRMS and MS/MS spectra of *N,N*-dimethyl-13-dihydrodaunorubicin (10) from crude extract.

HRMS **(A)** and MS/MS **(B)** spectra were obtained from a crude extract of MAG304.

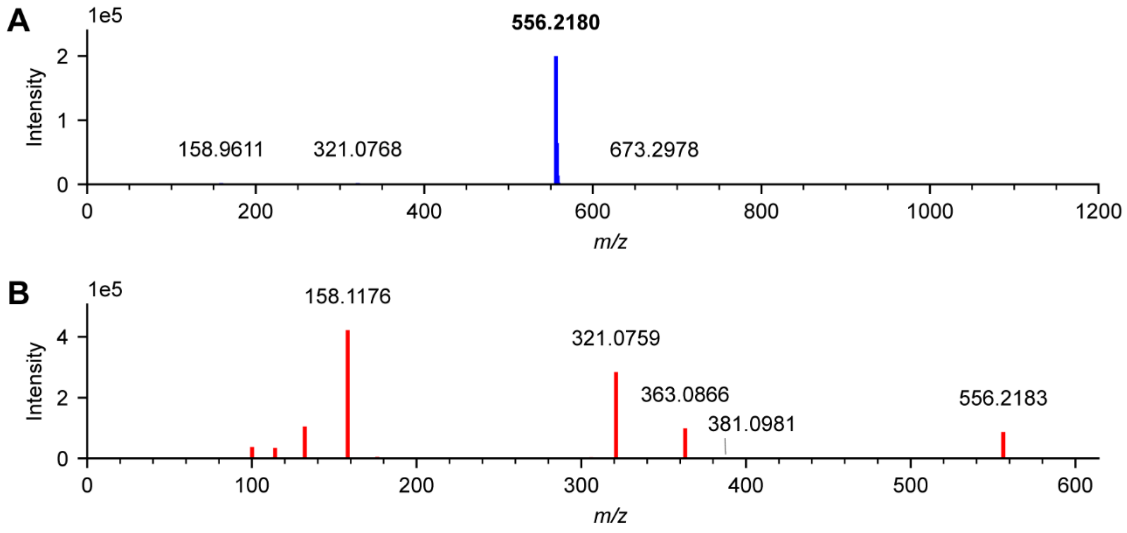

Figure S13. HRMS and MS/MS spectra of *N,N*-dimethyldaunorubicin (11) reference compound.

HRMS **(A)** and MS/MS **(B)** spectra were obtained from a reference compound of *N,N*-dimethyldaunorubicin (**11**).

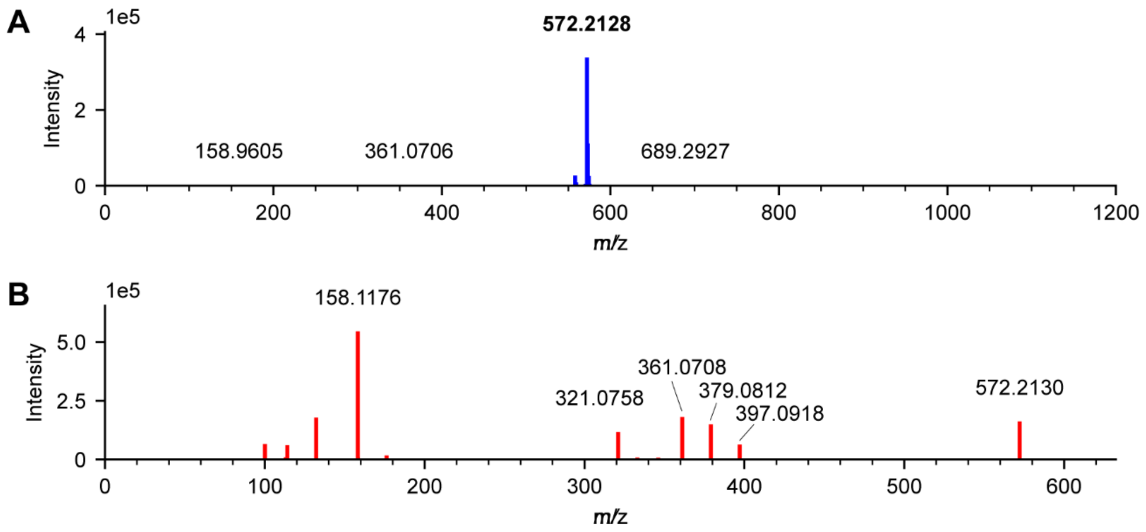

Figure S14. HRMS and MS/MS spectra of *N,N*-dimethyl-doxorubicin (12) reference compound.

HRMS **(A)** and MS/MS **(B)** spectra were obtained from a reference compound of *N,N*-dimethyldoxorubicin (**12**).

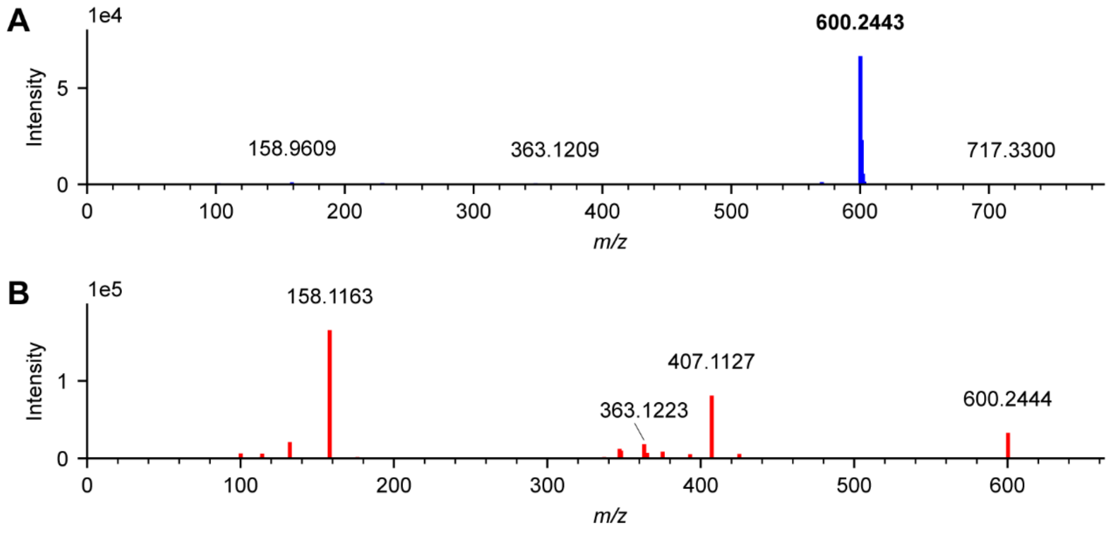

Figure S15. HRMS and MS/MS spectra of 4-methoxy-ε-rhodomycin T (13) from crude extract.

HRMS **(A)** and MS/MS **(B)** spectra were obtained from a crude extract of MAG301.

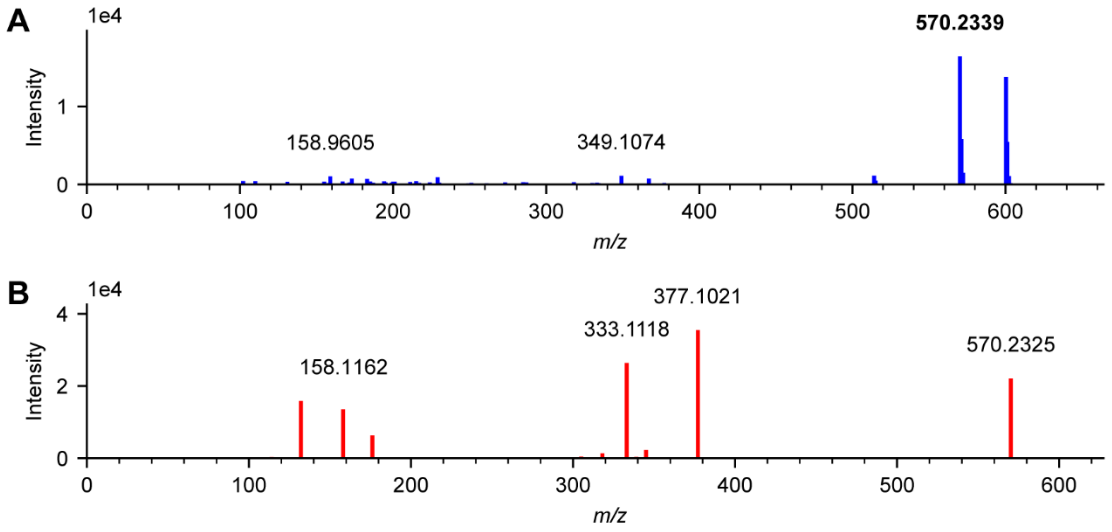

Figure S16. HRMS and MS/MS spectra of aclacinomycin T (14) from crude extract.

HRMS **(A)** and MS/MS **(B)** spectra were obtained from a crude extract of MAG301.

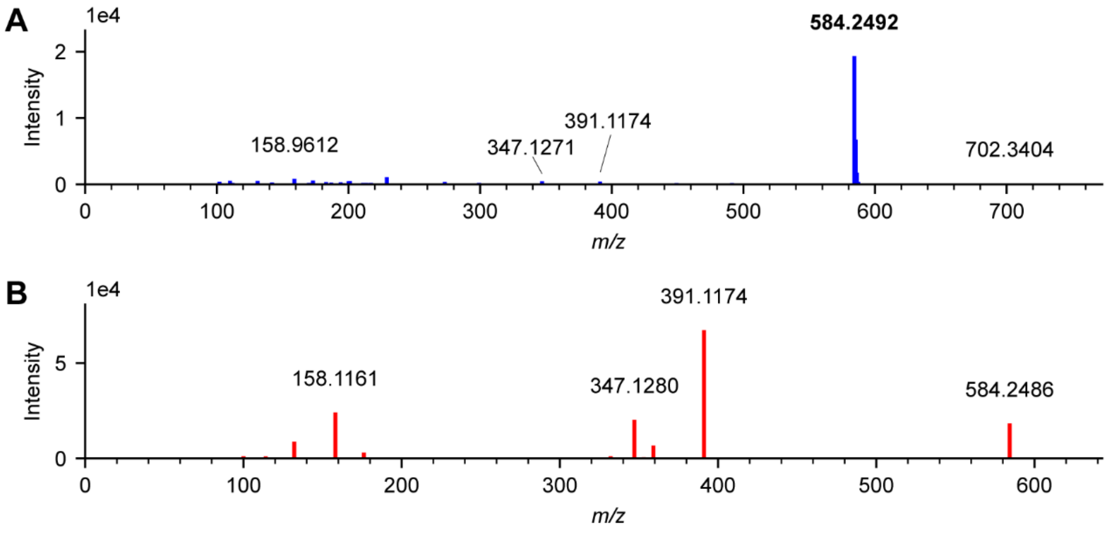

Figure S17. HRMS and MS/MS spectra of 4-methoxy-aclacinomycin T (15) from crude extract.

HRMS **(A)** and MS/MS **(B)** spectra were obtained from a crude extract of MAG301.

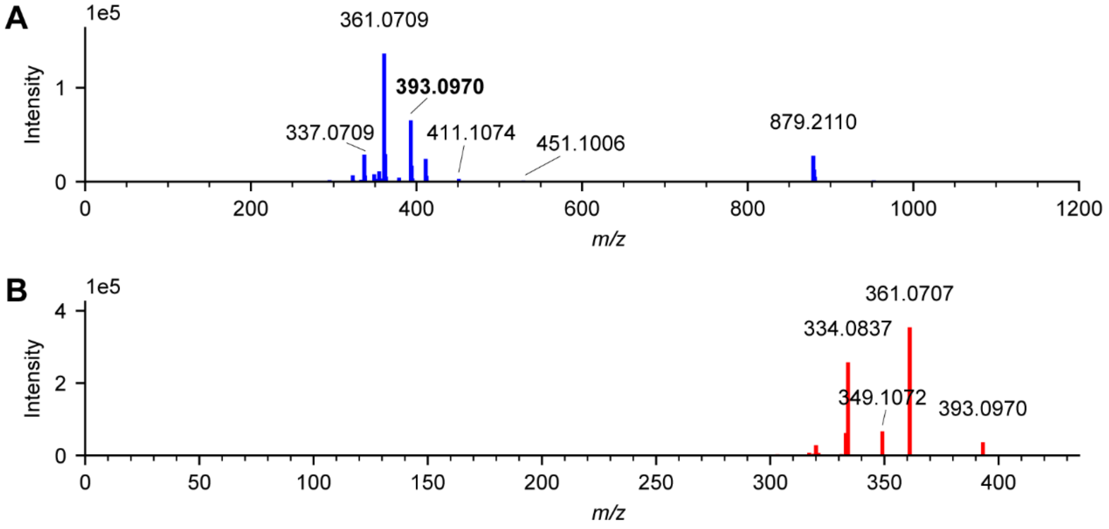

Figure S18. HRMS and MS/MS spectra of ε-rhodomycinone (16) from crude extract.

HRMS **(A)** and MS/MS **(B)** spectra were obtained from a crude extract of G001.

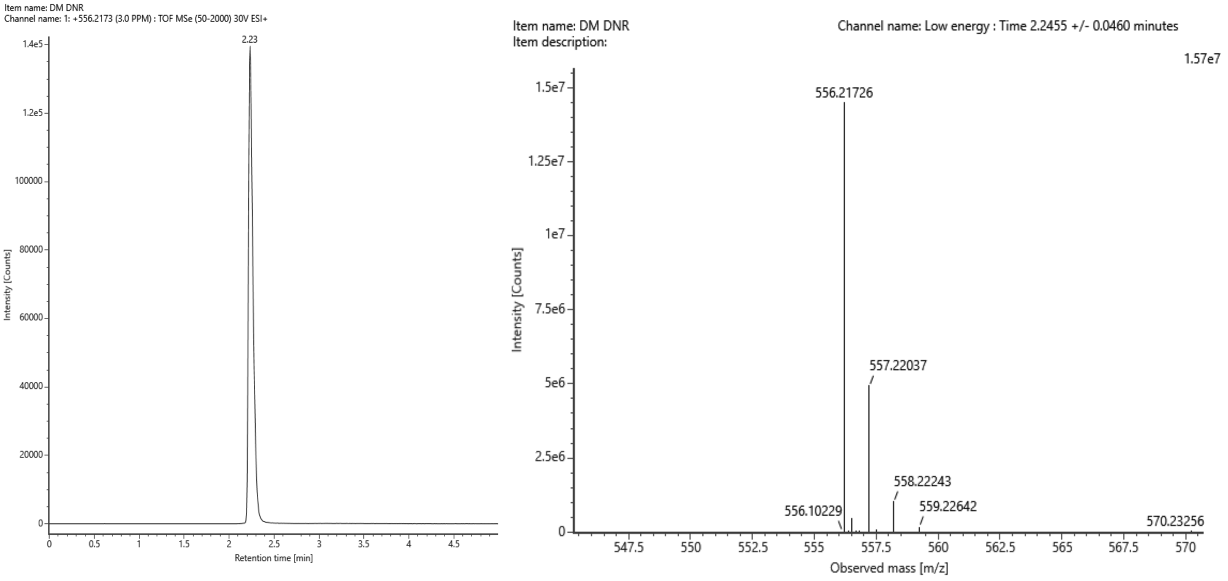

Figure S19. HRMS spectrum of *N,N*-dimethyldaunorubicin (11) reference compound using enzymatic assay LC-MS method.

(+)-HRESI-MS spectrum of *N*,*N*-dimethyldaunorubicin (**11**) reference compound. Calculated $[M+H]^+$ = 556.2177, measured $[M+H]^+$ = 556.2173.

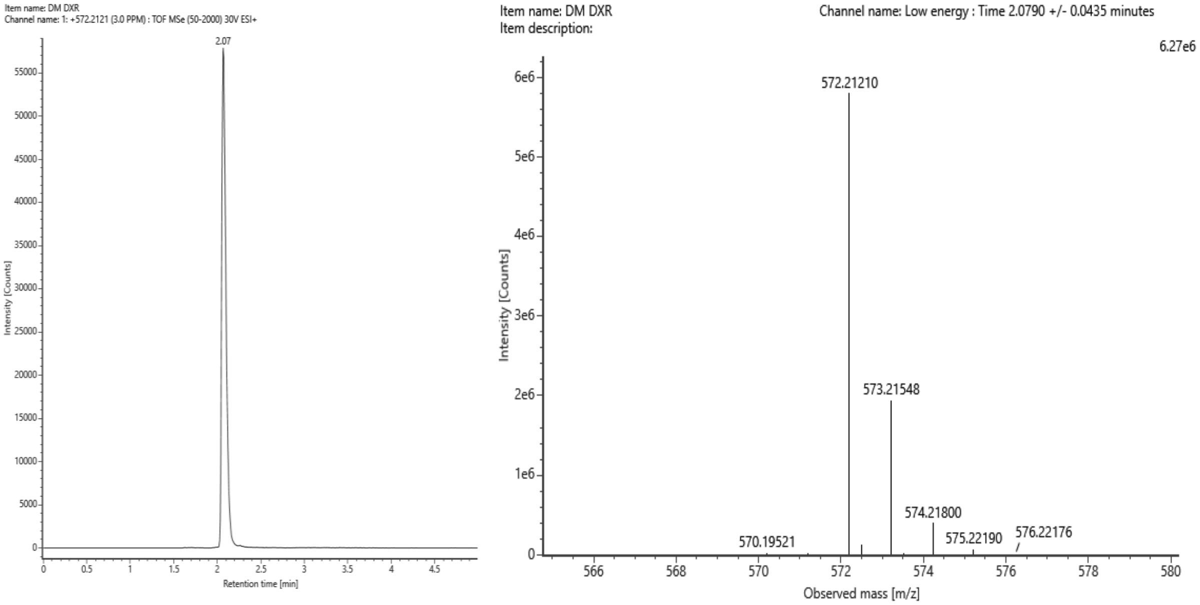

Figure S20. HRMS spectrum of *N,N*-dimethyldoxorubicin (12) reference compound using enzymatic assay LC-MS method.

(+)-HRESI-MS spectrum of *N*,*N*-dimethyldoxorubicin (**12**) reference compound using enzymatic assay LC-MS method. Calculated $[M+H]^+$ = 572.2126, measured $[M+H]^+$ = 572.2121.

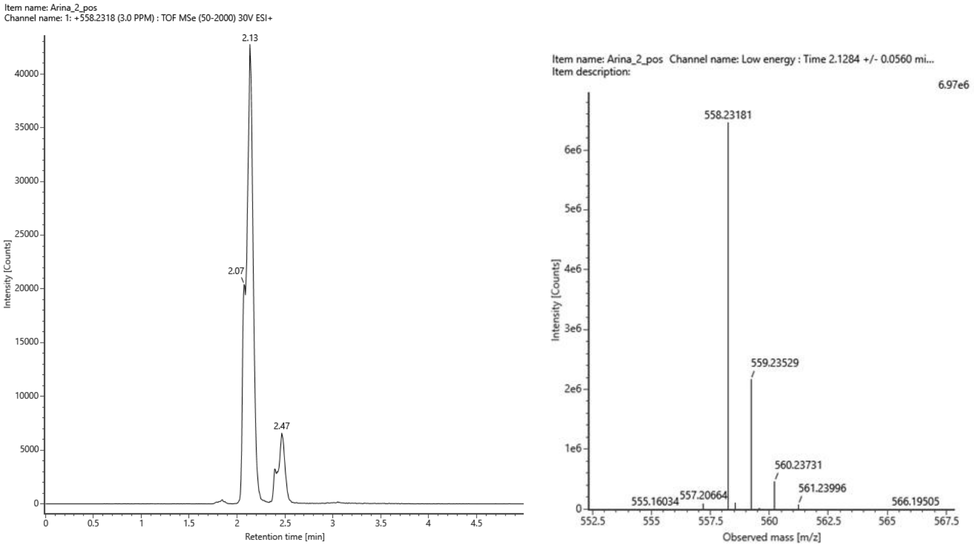

Figure S21. HRMS spectrum enzymatic assay of DoxA with *N*,*N*-dimethyl-13-deoxydaunorubicin (9).

(+)-HRESI-MS spectrum obtained from an enzymatic assay of DoxA with substrate *N*,*N*-dimethyl-13-deoxydaunorubicin (**9**). Reaction product *N*,*N*-dimethyl-13-dihydrodaunorubicin (**10**): calculated $[M+H]^+$ = 558.2334, measured $[M+H]^+$ = 558.2318.

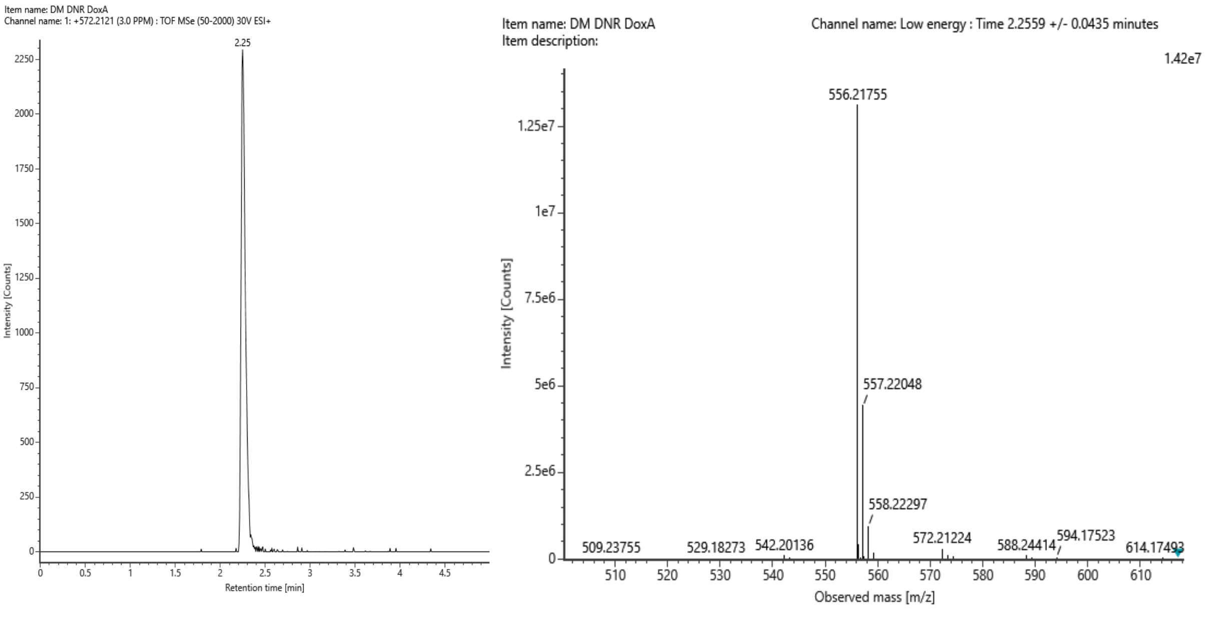

Figure S22. HRMS spectrum of enzymatic assay of DoxA with *N*,*N*-dimethyldaunorubicin (11).

(+)-HRESI-MS spectrum obtained from an enzymatic assay of DoxA with substrate *N*,*N*-dimethyldaunorubicin (**11**). Expected reaction product *N*,*N*-dimethyldoxorubicin (**12**): calculated $[M+H]^+$ = 572.2126. This ion was not detected at the retention time of the reference compound (2.08 min) (see Figure S20).

3 References

Plasmid cloning vectors for the conjugal transfer of DNA from *Escherichia coli* to *Streptomyces* spp. *Gene* 116, 43–49. doi: 10.1016/0378-1119(92)90627-2.

Chen, Y.-J., Liu, P., Nielsen, A. A. K., Brophy, J. A. N., Clancy, K., Peterson, T., et al. (2013). Characterization of 582 natural and synthetic terminators and quantification of their design constraints. *Nat. Methods* 10, 659–664. doi: 10.1038/nmeth.2515.

Fedoryshyn, M., Welle, E., Bechthold, A., and Luzhetskyy, A. (2008). Functional expression of the Cre recombinase in actinomycetes. *Appl. Microbiol. Biotechnol.* 78, 1065–1070. doi: 10.1007/s00253-008-1382-9.

Gregory, M. A., Till, R., and Smith, M. C. M. (2003). Integration site for *Streptomyces* phage φBT1 and development of site-specific integrating vectors. *J. Bacteriol.* 185, 5320–5323. doi: 10.1128/JB.185.17.5320-5323.2003.

Grocholski, T., Dinis, P., Niiranen, L., Niemi, J., and Metsä-Ketelä, M. (2015). Divergent evolution of an atypical *S*-adenosyl-L-methionine–dependent monooxygenase involved in anthracycline biosynthesis. *Proc. Natl. Acad. Sci. U.S.A.* 112, 9866–9871. doi: 10.1073/pnas.1501765112.

Han, A. R., Park, J. W., Lee, M. K., Ban, Y. H., Yoo, Y. J., Kim, E. J., et al. (2011). Development of a *Streptomyces venezuelae*-based combinatorial biosynthetic system for the production of glycosylated derivatives of doxorubicin and its biosynthetic intermediates. *Appl. Environ. Microbiol.* 77, 4912–4923. doi: 10.1128/AEM.02527-10.

Koroleva, A., Artukka, E., Yamada, K., Ilomäki, M., Kannisto, M., Wander, D. P. A., et al. (2024). Metabolic engineering for production of biosynthetic doxorubicin, manuscript under preparation.

Qiao, X., van der Zanden, S. Y., Wander, D. P. A., Borràs, D. M., Song, J.-Y. Y., Li, X., et al. (2020). Uncoupling DNA damage from chromatin damage to detoxify doxorubicin. *Proc. Natl. Acad. Sci. U.S.A.* 117, 15182–15192. doi: 10.1073/pnas.1922072117.

van Gelder, M. A., van der Zanden, S. Y., Vriends, M. B. L., Wagensveld, R. A., van der Marel, G. A., Codée, J. D. C., et al. (2023). Re-exploring the anthracycline chemical space for better anti-cancer compounds. *J. Med. Chem.* 66. doi: 10.1021/acs.jmedchem.3c00853.

Vara, J., Lewandowska-Skarbek, M., Wang, Y. G., Donadio, S., and Hutchinson, C. R. (1989). Cloning of genes governing the deoxysugar portion of the erythromycin biosynthesis pathway in *Saccharopolyspora erythraea* (*Streptomyces erythreus*). *J. Bacteriol.* 171, 5872–5881. doi: 10.1128/jb.171.11.5872-5881.1989.

Wu, C., van der Heul, H. U., Melnik, A. V., Lubben, J., Dorrestein, P. C., Minnaard, A. J., et al. (2019). Lugdunomycin, an angucycline-derived molecule with unprecedented chemical architecture. *Angew. Chem. Int. Ed.* 58, 2809–2814. doi: 10.1002/anie.201814581.
